# Bioactive fluorenes. Part III: 2,7-dichloro-9*H*-fluorene-based thiazolidinone and azetidinone analogues as anticancer and antimicrobial against multidrug resistant strains agents

**DOI:** 10.1186/s13065-020-00694-2

**Published:** 2020-06-25

**Authors:** Essam M. Hussein, Reem I. Alsantali, Moataz Morad, Rami J. Obaid, Hatem M. Altass, Ali Sayqal, Mohamed A. S. Abourehab, Amal A. Elkhawaga, Ahmed S. M. Aboraia, Saleh A. Ahmed

**Affiliations:** 1 Department of Chemistry, Faculty of Applied Science, Umm Al-Qura University, Makkah, 21955 Saudi Arabia; 2 Chemistry Department, Faculty of Science, Assiut University, Assiut, 71516 Egypt; 3 Department of Pharmaceutical Chemistry, Pharmacy College, Taif University, 888, Taif, Saudi Arabia; 4 Department of Pharmaceutics, Faculty of Pharmacy, Umm Al-Qura University, Makkah, Saudi Arabia; 5 Department of Pharmaceutics and Industrial Pharmacy, Faculty of Pharmacy, Minia University, Minia, Egypt; 6 Department of Medical Microbiology & Immunology, Faculty of Medicine Assiut University, Assiut, 71516 Egypt; 7 Department of Medicinal Chemistry, Faculty of Pharmacy, Assiut University, Assiut, 71516 Egypt

**Keywords:** Fluorene, Thiazolidinones, Azetidinones, Pharmacophores, Antimicrobial, Anti-cancer, FACS, Molecular docking

## Abstract

**Background:**

Thiazoles, thiazolidinones and azetidinones are highly ranked amongst natural and synthetic heterocyclic derivatives due to their great pharmaceutical potential.

**Results:**

New thiazolidinone and azetidinone class of bioactive agents based on 4-(2,7-dichloro-9*H*-fluoren-4-yl)thiazole moiety have been successfully synthesized. 4-(2,7-dichloro-9*H*-fluoren-4-yl)thiazol-2-amine was synthesized and allowed to react with various aryl/heteroaryl aldehydes to afford the corresponding Schiff base intermediates. The target thiazolidinone and azetidinone analogues have derived from Schiff bases by their reactions with thioglycolic acid and chloroacetyl chloride, respectively. The newly synthesized compounds were then evaluated for their antimicrobial activity against some multidrug resistant strains and examined for cytotoxic activity against normal lung fibroblast (WI-38), human lung carcinoma (A549), and human breast carcinoma (MDA-MB-231) cell lines to develop a novel class of fluorene-based bioactive agents. The mode of action and the binding interaction of the synthesized compound with the active sites of dihydrofolate reductase enzyme were well identified by fluorescence-activated cell sorting (FACS) analysis and molecular docking study.

**Conclusion:**

Some of the synthesized compounds showed remarkable activity against A-549 and MDA-MB-231 when compared to Taxol, which was used as a reference drug. 2,7-dichloro-9*H*-fluorene-based azetidinones are more efficient as antimicrobial and anticancer agents compared to dichloro-9*H*-fluorene-based thiazolidinones derivatives.
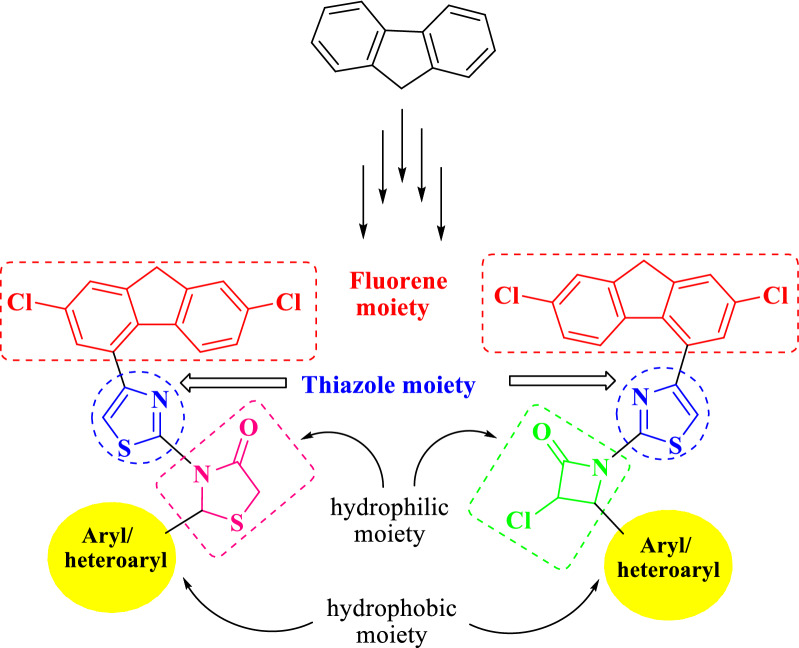

## Introduction

**Fig. 1:**
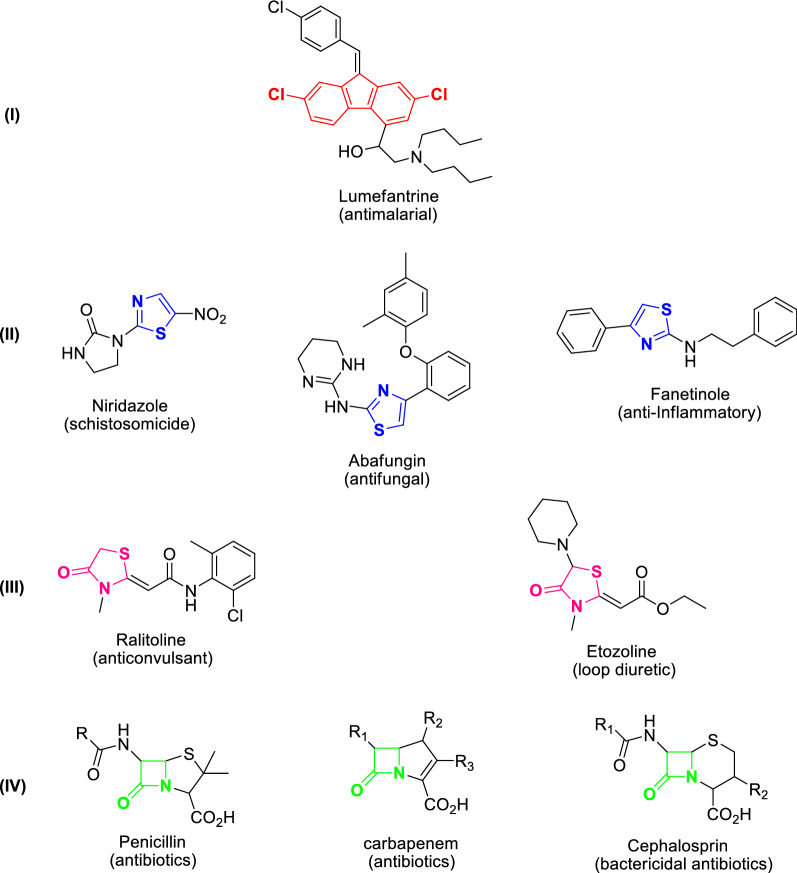
Representative examples of drugs containing 2,7-dichloro-9*H*-fluorene (I), thiazole (II), 4-thiazolidinone (III) and 2-azetidinone (IV) moieties

In the last few years, fluorene derivatives exposed effective uses as precursors in broad ranging of synthetic and medical applications [1]. As example, 2,7-dichloro-7*H*-fluorene considered as a backbone moiety for the synthesis of a well-known antimalarial drug which known as Lumefantrine [2] (Fig. 1). On the other hand, heterocyclic compounds are highly ranked amongst natural and synthetic pharmaceutically significant agents. The fabulous ability of heterocyclic moiety to serve as both biomimetic and active pharmacophores has mainly contributed to their distinctive value as traditional key elements of various drugs. Due to their broad pharmacological profile, the nitrogen and sulfur-containing heterocycles demonstrate an imperative class in the biological research and drug industry areas [3–8]. Amongst them, the thiazole ring is a core structural moiety found in a wide range of biologically and medicinally active molecules. The thiazole derivatives are useful for treatment of several diseases such as allergies [9], hypertension [10], microbial [11], human immunodeficiency virus (HIV) infections [12], inflammation [13], and schizophrenia [14]. Moreover, 4-thiazolidinone and its derivatives have considerable attention for the last decades due to their pharmacological potential. These derivatives are known to acquire several promising chemotherapeutical activities such as antihistaminic [15], anti-inflammatory [16], hypolipidaemic [17], antimicrobial [18], anticonvulsant and antipsychotic [19], antimalarial [20], and anti-cancer [21] activities. Numerous drugs containing thiazole or 4-thiazolidinone moieties in their structure used in broad range in the pharmaceutical market such as Niridazole, Abafungin, Fanetinole, Ralitoline and Etozoline (Fig. 1). The traditional synthesis of 4-thiazolidinone derivatives involves cycloaddition of Schiff base with thioglycolic acid [22]. Additionally, the 2-azetidinone moiety is commonly show wide range of biological activities and exist in several *β*-lactam antibiotics such as penicillins, carbapenems and cephalosporins (Fig. 1) which are used as broad spectrum antibacterial agents. A large number of 3-chloro monocyclic β-lactam exhibits powerful antimicrobial, anticonvulsant, anti-inflammatory and antitubercular activities [23–25]. Conventional synthesis of 3-chloro-2-azetidinones involves [2 + 2] Staudinger’s ketene-imine cycloaddition reaction between chloroacetyl chloride and Schiff bases [26].

On the other hand, dihydrofolate reductase (DHFR) is an indispensable enzyme that catalyzes the NADPH-dependent reduction of 7,8-dihydrofolate (DHF) to 5,6,7,8-tetrahydrofolate (THF), which is the precursor of the cofactors compulsory for the biosynthesis of thymidine and purine nucleotides [27]. Accordingly, inhibition of dihydrofolate reductase lead to the disturbance of deoxyribonucleic acid (DNA) synthesis and the death of the proliferating cells [27, 28]. Furthermore, bacteria need DHFR to grow and multiply and consequently inhibitors discerning for bacterial in contradiction of host DHFR have found usage as antibacterial agents [29]. These two remarkable features render DHFR enzyme as one of the main targets for both antimicrobial and anticancer drug design [30, 31].

In the light of the previous findings, we predicted that the combining of 2,7-dichlorofluorene moiety with the versatile thiazole, thiazolidinone and azetidinone pharmacophores into a single chemical structure could be competent for antimicrobial and anticancer activities [30–34]. As part of our interest towards the development of novel bioactive organic molecules [30–34], a drug strategy has been planned to synthesis of some novel 2-(aryl/heteroaryl)-3-(4-(2,7-dichloro-9*H*-fluoren-4-yl)thiazol-2-yl)thiazolidin-4-ones and 3-chloro-4-(aryl/heteroaryl)-1-(4-(2,7-dichloro-9*H*-fluoren-4-yl)thiazol-2-yl)azetidin-2-ones with the anticipation to improve the antimicrobial activity against multidrug resistant strains and anticancer activity against human lung carcinoma (A549), and human breast carcinoma (MDA-MB-231) cell lines.

## Results and discussion

### Chemistry

**Fig. 2:**
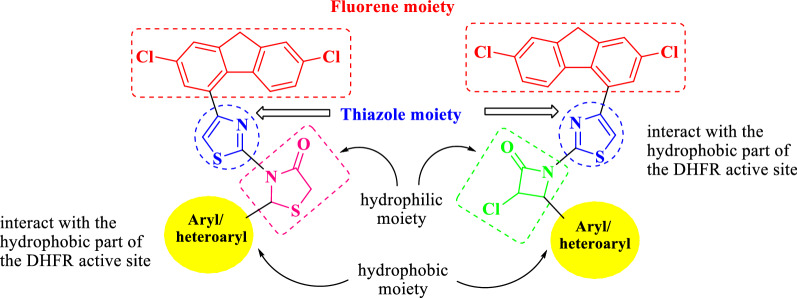
Structural fragments of DHFR inhibitors in the DHFR enzymatic active site

As the inhibition of DHFR is commonly considered as one of the most prominent mechanism in elucidating antimicrobial and anticancer activities [35, 36], the compounds synthetic approaches were designed in order to achieve: (i) possess hydrophilic and hydrophobic parts that can interact with the hydrophilic and hydrophobic regions of the DHFR active site, respectively; (ii) comply with the pharmacophores that may interest as DHFR inhibitors, as presented in Fig. 2.

**Scheme 1:**
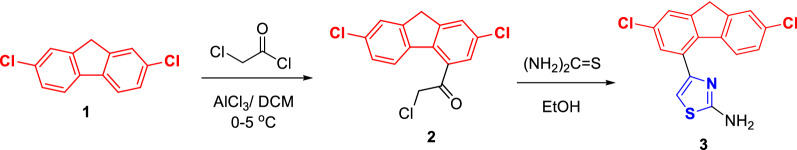
Synthesis of 4-(2,7-dichloro-9*H*-fluoren-4-yl)thiazol-2-amine (**3**)

**Scheme 2:**
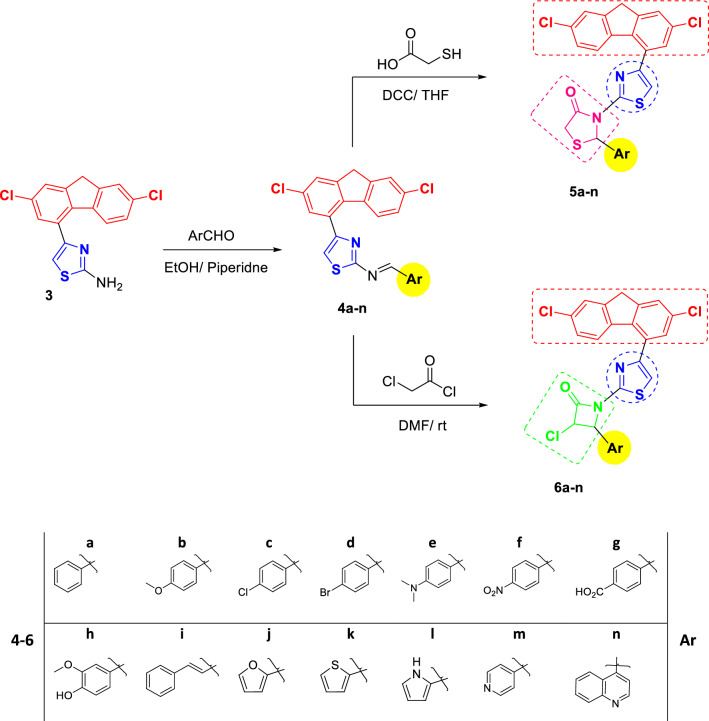
Synthesis of the target thiazolidinone derivatives **5a**–**n** and azetidinone derivatives **6a**–**n**

A distinctive synthetic approach employed to synthesize the target fluorene derivatives (**5**, **6**) in good yields is described in Schemes 1 and 2. The synthetic strategy starts with a simple and convenient methodology to 2-chloro-1-(2,7-dichloro-9*H*-fluoren-4-yl)ethanone (**2**) involving direct chloroacetylation of 2,7-dichloro-9*H*-fluoren (**1**) is performed in excellent yield by adding a solution of **1** in dichloromethane (DCM) at 0–5 °C to a suspension of chloroacetyl chloride and aluminum chloride in dichloromethane according to our previously reported procedure [31]. Accordingly, 4-(2,7-dichloro-9*H*-fluoren-4-yl)thiazol-2-amine (**3**) is attained in 97% yield via Hantzsch reaction of 2-chloro-1-(2,7-dichloro-9*H*-fluoren-4-yl)ethanone (**2**) with thiourea in refluxing ethanol (Scheme 1).

4-(2,7-Dichloro-9*H*-fluoren-4-yl)thiazol-2-amine (**3**) on condensation with different aryl/heteroaryl aldehydes in ethanol using catalytic amount of piperidine under reflux conditions afforded 4-(2,7-dichloro-9*H*-fluoren-4-yl)-*N*-(aryl/heteroaryl-methylene)thiazol-2-amine (**4a**–**n**) in 71–96% yields. Cyclocondensation of compounds (**4a**–**n**) with thioglycolic acid in tetrahydrofuran (THF) in presence of *N*,*N*′-dicyclohexylcarbodiimide (DCC) as a dehydrating agent under reflux conditions yielded the target 2-(aryl/heteroaryl)-3-(4-(2,7-dichloro-9*H*-fluoren-4-yl)thiazol-2-yl)thiazolidin-4-ones (**5a**–**n**) in 64–90% yields. Moreover, **4a**–**n** when subjected to cyclocondensation with chloroacetyl chloride in dimethylformamide (DMF) at room temperature, 3-chloro-4-(aryl/heteroaryl)-1-(4-(2,7-dichloro-9*H*-fluoren-4-yl)thiazol-2-yl)azetidin-2-ones (**6a**–**n**) were obtained in moderate to excellent yields (51–98%) (Scheme 2).

The chemical structures of all synthesized compounds **5a**–**n** and **6a**–**n** were well-confirmed based on spectroscopic data such as Fourier transform infrared (FT**-**IR), proton nuclear magnetic resonance (^1^H-NMR), carbon-13 nuclear magnetic resonance (^13^C-NMR) and The distortionless enhancement by polarization transfer (DEPT-135) data (c.f. “Experimental” section and Additional file 1). The FT-IR spectra of compounds **5a**–**n** revealed the presence of characteristic absorption bands at 1780–1680 cm^−1^ for (C=O) group, 1636–1600 cm^−1^ for (C=N) group. Furthermore, to fully establish the chemical structures of the products, intensive 1D (^1^H, ^13^C, and DEPT-135) NMR spectroscopic analysis were recorded. For example, analysis of the ^13^C and ^13^C-DEPT-135 NMR spectra of **5a** indicated the presence of 23 signals representing the 23 of nonequivalent carbons (10 aromatic quaternary carbons, 9 aromatic CH’s, 2 methylene carbons, one methine carbon and one carbonyl carbon). Its ^1^H-NMR spectrum showed three singlet signals at 7.66, 7.60, 7.28 ppm and two doublets at 7.48 and 6.99 ppm (*J* = 8.0 Hz) for five protons of the fluorene moiety. A multiplet at 7.20, 6.94 ppm and doublet signals at 7.38, 6.27 ppm (*J* = 8.0 Hz) appeared for the protons of phenyl moiety. In addition to this, a singlet signal at 6.76 ppm for thiazole moiety. Three singlet signals at 3.98, 3.60 and 3.51 ppm corresponded to two methylene and one methine protons.

On the other hand, the FT-IR spectra of compounds **6a**–**n** showed the presence of characteristic absorption bands at 1792–1697 cm^−1^ for (C=O) group, 1698–1598 cm^−1^ for (C=N) group. Indeed, the ^13^C and ^13^C-DEPT-135 NMR spectra of **6b** indicated the presence of 24 signals representing the 24 of nonequivalent carbons (11 aromatic quaternary carbons, 8 aromatic CH’s, 2 methine carbons, one methylene carbon, one methyl carbon and one carbonyl carbon). Its ^1^H-NMR spectrum showed two doublets at 7.87 and 7.27 ppm (*J* = 8.0, 8.0 Hz), three singlet signals at 7.70, 7.50 and 7.21 ppm for five protons of the fluorene moiety. Two multiplets at 7.64 and 7.44 ppm appeared for the protons of 4-methoxyphenyl moiety. In addition, a singlet signal at 7.12 ppm for thiazole moiety. Four singlet signals at 4.45, 4.28, 4.20, and 4.01 ppm corresponded to the one methyl, two methine and one methylene protons.

### Biological activity

#### Antimicrobial activity

Nowadays, the microbial resistance to currently found antibiotics is considered a precarious problem. Therefore, performing some more trials and efforts to identify novel targets for discovering new antibiotics is supposed to be a strong challenge [37]. The multidrug resistant bacteria have been reported with a diversity of nosocomial and community acquired infections as pneumonia, surgical site infections and urinary tract infections [38].

In the current study, the synthesized fluorene derivatives **5a**–**n** and **6a**–**n** were evaluated for their antimicrobial activity against multidrug resistant strains of Gram-positive bacteria such as *staphylococcus aureus* (*S. aureus*), methicillin-resistant *Staphylococcus aureus* (*MRSA*) and *Streptococcus pneumoniae* (*S. pneumoniae*) and Gram-negative bacteria such as *Escherichia coli* (*E. coli*), *Klebsiella pneumoniae* (*K. pneumoniae*), *Pseudomonas aeruginosa* (*P. aeruginosa*) and *Acinetobacter baumannii* (*A. baumannii*) as well as three fungal strains such as *Aspergillus flavus* (*A. flavus*), *Aspergillus niger* (*A. niger*) and *Candida albicans* (*C. albicans*). Screening the antimicrobial activity was done by agar well diffusion assay [39] using a concentration of 500 µg/mL of the tested fluorene compounds, the results of the antimicrobial assay are given in Tables 1, 2, 3. It is clearly observed that some of the newly synthesized fluorene derivatives exhibited comparatively high antimicrobial activity when compared to the positive reference drugs; *vancomycin* for Gram-positive bacteria, *gentamicin* for Gram-negative bacteria and *fluconazole* for fungi. It’s worthy to mention that, the thiazolidinone derivatives **5g**, **5h**, **5i** and **5l** produced relative high activity against *S. aureus* with a zone of inhibition (ZOI) value 10 mm, 11 mm, 10 mm, and 9 mm, respectively. While the compound **5j** showed higher activity against *E. coli* and *P. aeruginosa* with a zone of inhibition (ZOI) value 10 mm and 8 mm, respectively (Table 1).

**Table 1:** Antimicrobial activity of the newly synthesized thiazolidinone derivatives **5a**–**n** against the multidrug resistant tested microbial strains

Comp.	Gram (+ve) bacteria			Gram (−ve) bacteria				Fungi		
	*S. aureus*	MRSA	*S. pneumoniae*	*E. coli*	*K. pneumoniae*	*P. aeruginosa*	*A. baumannii*	*A. flavus*	*A. niger*	*C. albicans*
**5a**	3	–	–	–	–	–	–	–	–	–
**5b**	4	–	–	–	–	–	–	–	–	–
**5c**	3	–	–	–	–	–	–	–	–	–
**5d**	4	–	–	–	–	–	–	–	–	–
**5e**	–	–	–	–	–	–	–	–	–	–
**5f**	–	–	–	–	–	–	–	–	–	–
**5g**	10	–	–	–	–	–	–	–	–	–
**5h**	11	–	–	–	–	–	–	–	–	–
**5i**	10	–	–	–	–	–	–	–	–	–
**5j**	6	–	–	10	–	8	–	–	–	–
**5k**	–	–	–	–	–	–	–	–	–	–
**5l**	9	–	–	6	–	–	–	–	–	–
**5m**	5	–	–	8	–	–	–	–	–	–
**5n**	7	–	–	–	5	–	–	–	–	–
**PC**	28	26	20	28	20	30	20	18	18	20

Mean zone of inhibition in mm, –  resistant

*PC* positive control (*Vancomycin* 50 µg/mL for Gram-positive bacteria and *Gentamicin* 10 µg/mL for Gram-negative bacteria), *fluconazole* 25 µg/mL for fungi

**Table 2:** Antimicrobial activity of the newly synthesized azetidinone derivatives **6a**–**n** against the multidrug resistant tested microbial strains

Comp.	Gram (+ve) bacteria			Gram (−ve) bacteria				Fungi		
	*S. aureus*	MRSA	*S. pneumoniae*	*E. coli*	*K. pneumoniae*	*P. aeruginosa*	*A. baumannii*	*A. flavus*	*A. niger*	*C. albicans*
**6a**	5	–	–	13	–	–	–	–	–	–
**6b**	–	–	–	12	–	–	–	–	–	–
**6c**	7	–	–	15	6	10	–	–	–	–
**6d**	3	–	–	5	5	8	–	–	–	–
**6e**	12	–	–	–	–	–	–	–	–	–
**6f**	10	–	–	–	–	–	5	–	–	–
**6g**	–	–	–	–	–	–	–	–	–	–
**6h**	12	15	–	15	3	–	–	–	–	–
**6i**	–	–	–	12	3	–	–	–	–	–
**6j**	9	–	–	20	–	–	6	–	–	–
**6k**	7	–	–	14	3	11	–	–	–	–
**6l**	15	15	–	22	–	8	–	–	–	–
**6m**	4	6	–	27	3	–	–	–	–	–
**6n**	9	–	–	19	5	13	5	–	–	–
**PC**	28	26	20	28	20	30	20	18	18	20

Mean zone of inhibition in mm, – = resistant

*PC* positive control (*Vancomycin* 50 µg/mL for Gram-positive bacteria and *Gentamicin* 10 µg/mL for Gram-negative bacteria), *fluconazole* 25 µg/mL for fungi

**Table 3:** Determination of minimum inhibitory concentration (MIC) of the most active newly synthesized fluorene derivatives **5a**–**n** and **6a**–**n**

Comp.	Tested strain/MIC (µg/mL)				
	*S. aureus*	MRSA	*E. coli*	*K. pneumoniae*	*P. aeruginosa*
**5h**	62.5	–	–	–	–
**5j**	–	–	62.5	–	62.5
**6c**	–	–	62.5	125	–
**6e**	62.5	–	–	–	–
**6h**	62.5	31.25	31.25	–	–
**6j**	–	–	31.25	–	–
**6k**	–	–	31.25	–	125
**6l**	31.25	31.25	31.25	–	125
**6m**	–	–	15.6	–	–
**6n**	–	–	31.25	–	62.5
*Gentamicin* 10 µg/mL	–	–	0.3	0.3	0.3
*Vancomycin* 50 µg/mL	0.7	0.7	–	–	–
DMSO	–	–	–	–	–

Furthermore, azetidinone derivatives **6a**–**n** achieved relatively high antimicrobial activity against both Gram positive and Gram-negative bacteria, particularly **6h** against *S. aureus*, MRSA, *E. coli* and *P. aeruginosa* with a ZOI value 12 mm, 15 mm, 22 mm, and 8 mm, respectively. However, a higher activity was shown against *E. coli* with ZOI value 27 mm for compound **6m**. On the other hand, **6d** showed moderate activity against Gram-negative bacteria *E. coli*, *K. pneumoniae* and *P. aeruginosa* with a ZOI value 15 mm, 6 mm, 10 mm, and 8 mm, respectively. However, low activity was shown against *S. aureus* and no activity was shown against both MRSA and *S. pneumoniae*. Moreover, the compound **6n** showed moderate antimicrobial activity against *E. coli* and *P. aeruginosa* with a ZOI value 19 mm and 13 mm, respectively. The rest of the newly synthesized fluorene derivatives display low antimicrobial activity therefore these derivatives have potential for further comprehensive studies (Table 2).

The minimum inhibitory concentration (MIC) of the most active newly synthesized fluorene derivatives was determined and reported in Table 3. The MIC varied within the range (500 µg/mL–7.8 µg/mL). Compounds **5h** and **6e** were potent against Gram positive bacteria particularly *S. aureus* with an MIC value 62.5 µg/mL. Also, **6h** was potent but against both *S. aureus* and MRSA with an MIC (62.5–31.25 µg/mL, respectively). Furthermore, a lower MIC was observed by the compound **6l** against both *S. aureus* and MRSA as the MIC value was (31.25 µg/mL). On the other hand, the newly synthesized fluorene derivatives showed higher activity against Gram negative bacteria which is clearly achieved by the compound **6j**, **6k**, **6l** and **6** **m** with MIC ranged from (31.25–15.6 µg/mL) specially against *E. coli*. All results were compared to vancomycin and Gentamicin as antibacterial reference drug (Table 3).

It’s worth to report that, the obtained biological activities make the newly synthesized novel fluorene derivatives **5a**–**n** and **6a**–**n**, interesting molecules for the synthesis of new antibiotics either alone or in combination with other compounds, and subsequently help in fighting the multidrug resistant superbugs.

#### In vitro anticancer activity

**Fig. 3:**
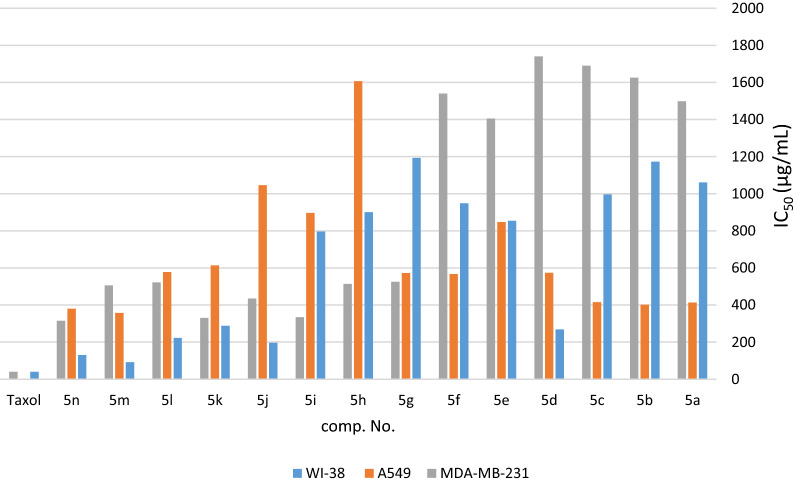
IC_50_ of the tested compounds **5a**–**n** against WI-38, A549, and MDA-MB-231 cancer cells after 24 h treatments

**Fig. 4:**
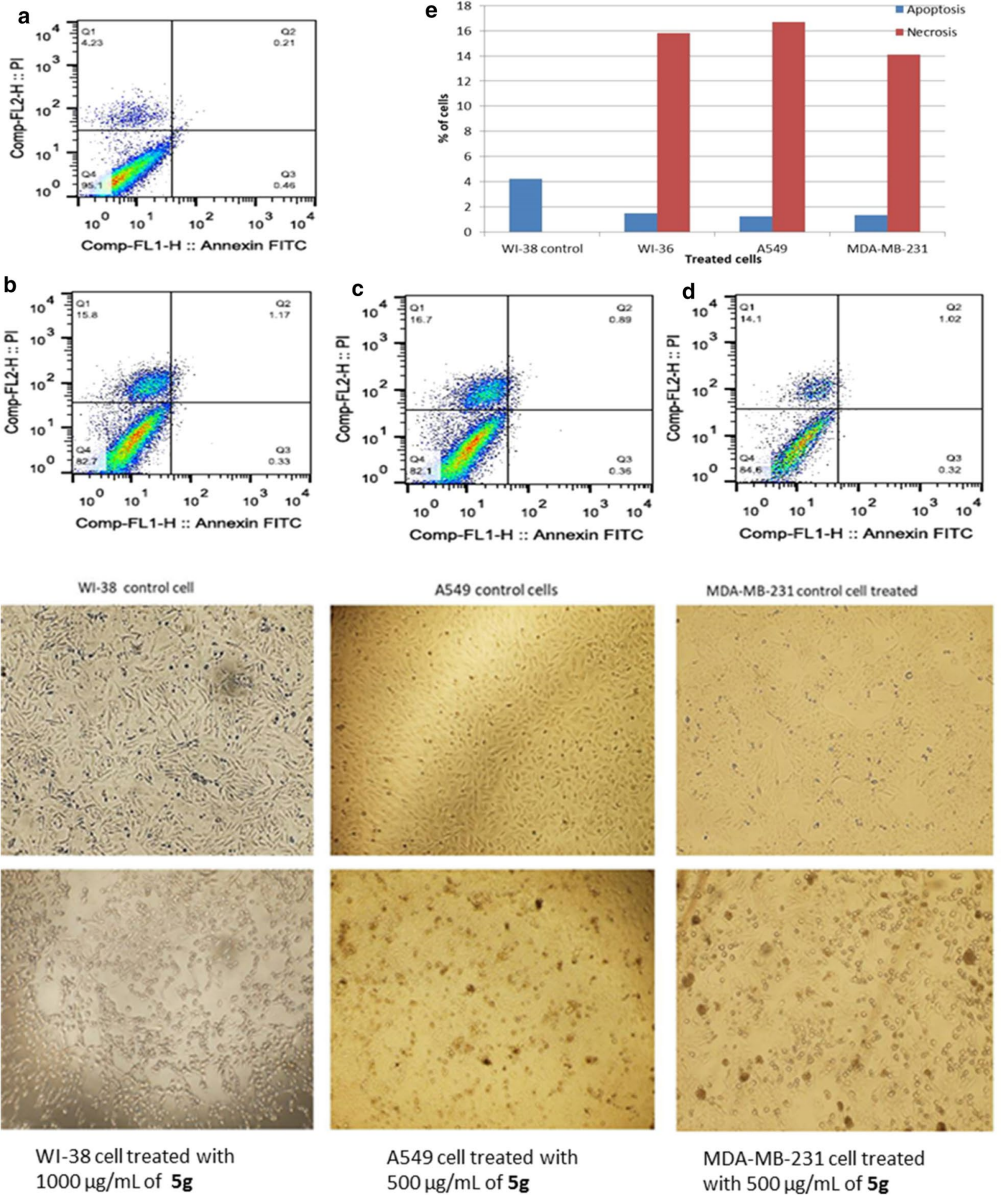
Apoptotic and necrotic cell death were assessed using Annexin V and Probidium Iodide (PI) staining and analyzed using flow cytometer after 24 h treatment with **5g**. **a** WI-38 cells control (DMSO), **b** WI-38 cell treated with 1000 µg/mL of **5g**, **c** A549 cell treated with 500 µg/mL of **5g**, **d** MDA-MB-231 cell treated with 500 µg/mL of **5g**, and (E) quantification of apoptotic and necrotic cell death for each drug on MDA-MB-231 cells

**Fig. 5:**
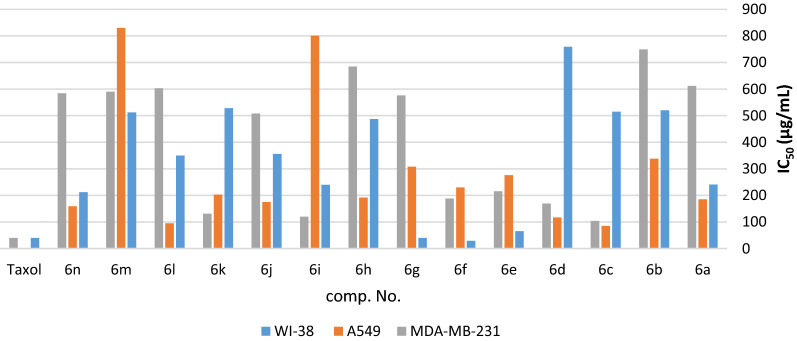
IC_50_ of the tested compounds **6a**–**n** against WI-38, A549, and MDA-MB-231 cancer cells after 24 h treatments

**Fig. 6:**
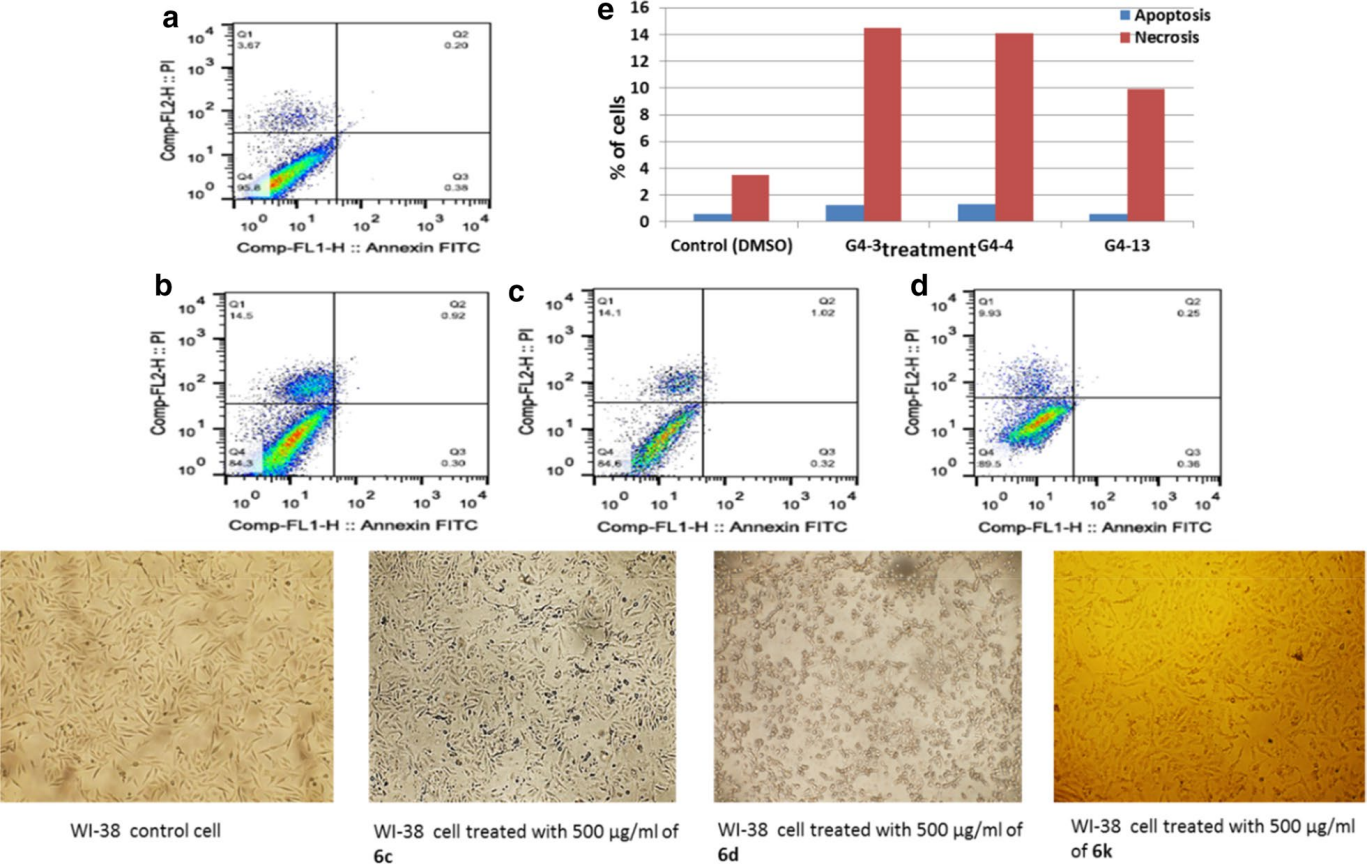
Apoptotic and necrotic cell death were assessed using Annexin V and Probidium Iodide (PI) staining and analyzed using flow cytometer after 24 h treatment of azetidinone derivatives. **a** WI-38 cells control (DMSO), **b** WI-38 cell treated with 500 µg/mL of **6c**, **c** WI-38 cell treated with 500 µg/mL of **6d**, **d** WI-38 cell treated with 500 µg/mL of **6k**, and **e** quantification of apoptotic and necrotic cell death for each drug on WI-38 cells

**Fig. 7:**
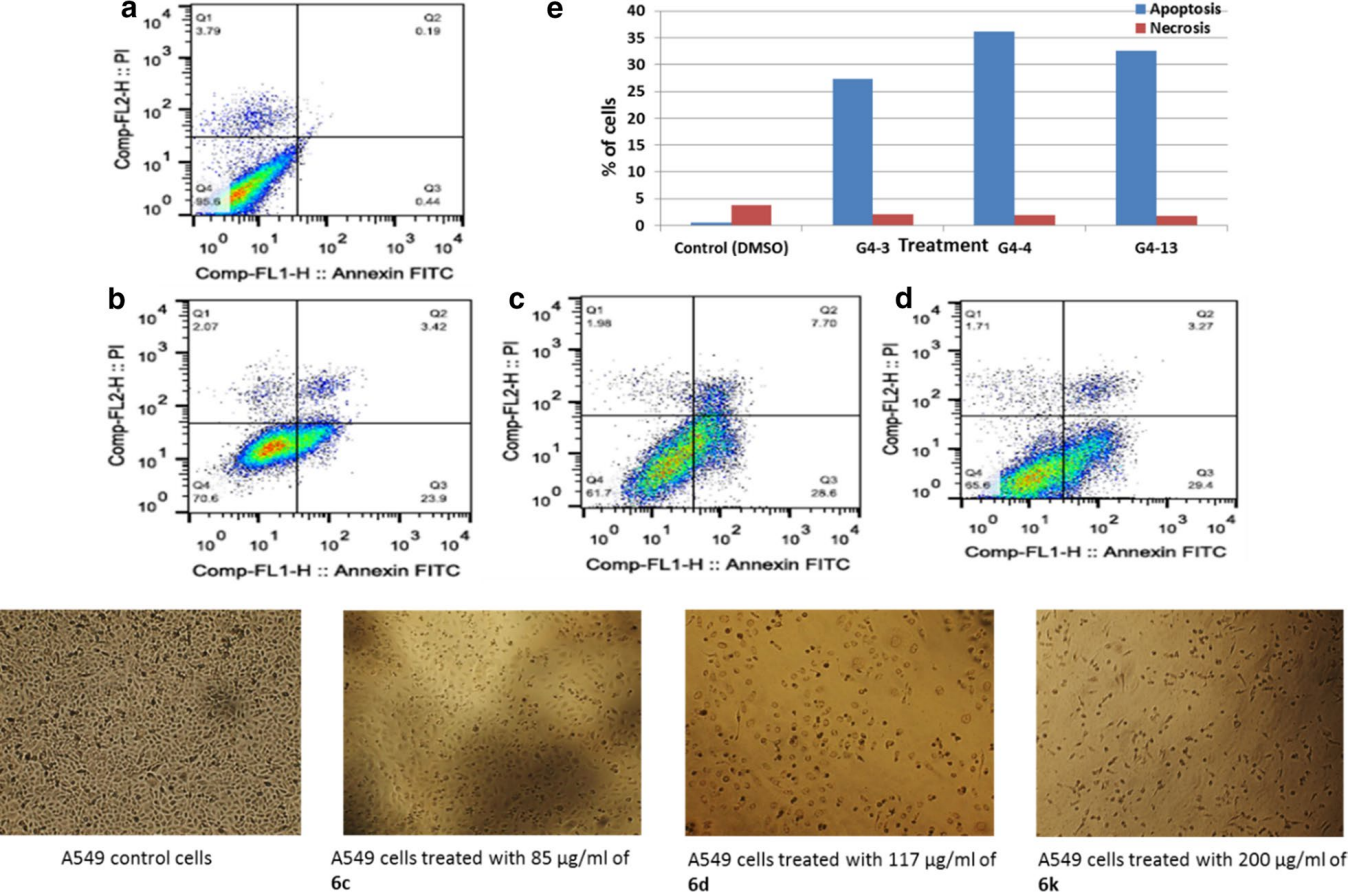
Apoptotic and necrotic cell death were assessed using Annexin V and Probidium Iodide (PI) staining and analyzed using flow cytometer after 24 h treatment with azetidinone derivatives. **a** A549 cells control in dimethyl sulphoxide (DMSO), **b** A549 cell treated with 85 µg/mL of **6c**, **c** A549 cell treated with 117 µg/mL of **6d**, **d** A549 cell treated with 200 µg/mL of **6k**, and **e** quantification of apoptotic and necrotic cell death for each drug on A549 cells

**Fig. 8:**
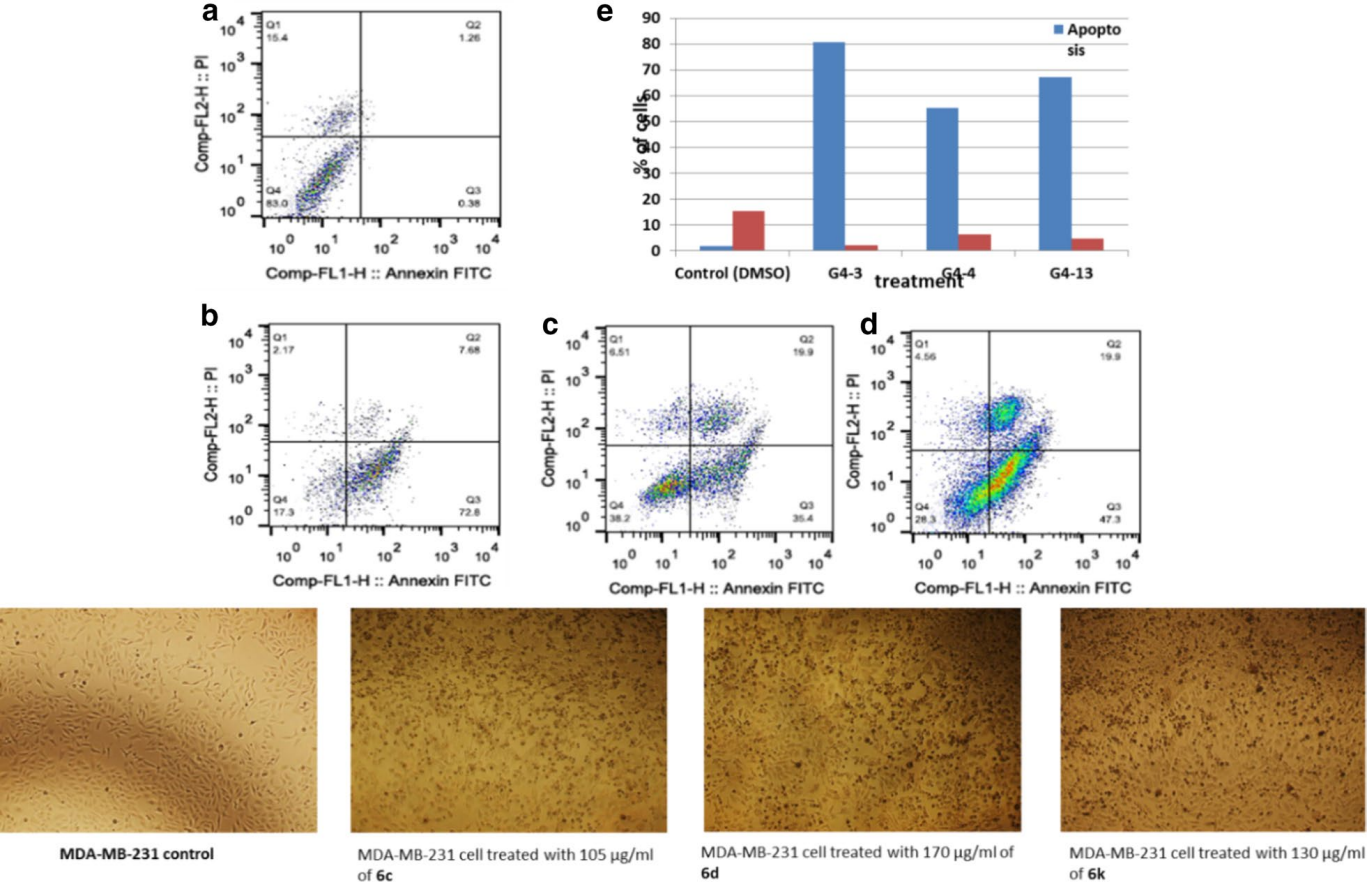
Apoptotic and necrotic cell death were assessed using Annexin V and Probidium Iodide (PI) staining and analyzed using flow cytometer after 24 h treatment with azetidinone derivatives. **a** MDA-MB-231 cells control (DMSO), **b** MDA-MB-231 cell treated with 105 µg/mL of **6c**, **c** MDA-MB-231 cell treated with 170 µg/mL of **6d**, **d** MDA-MB-231 cell treated with 130 µg/mL of **6k**, and **e** quantification of apoptotic and necrotic cell death for each drug on MDA-MB-231 cells

The synthesized new fluorene derivatives **5a**–**n** and **6a**–**n** were tested as anti-proliferative agents against WI-38 normal human lung fibroblast cells, A549 adenocarcinomic human alveolar basal epithelial cells, and MDA-MB-231 human breast cancer cells and they showed selectivity in their cytotoxic activity. A well-known chemotherapeutic agent, Taxol (IC_50_ = 41, 2.30, and 40 µg/mL for WI-38, A549, and MDA-MB-231, respectively) was used as reference control. The obtained results are presented in Tables 4, 5, 6, 7, 8, 9 and Figs. 3, 4, 5, 6, 7, 8.

**Table 4:** In vitro cytotoxic screening of **5a**–**n** against WI-38 human normal fibroblast cells

Comp.	Validity (%) for sample concentrations (µg/mL)						IC_50_ (µg/mL)
	0	31.25	62.50	125	250	500	
**5a**	100	89.8	83.3	80.6	77.3	74.3	1061
**5b**	100	99.4	94.4	91.3	81.2	79.00	1173
**5c**	100	89.7	87.3	83.8	77.5	71.3	996
**5d**	100	94.9	88.5	86.09	50.7	44.2	268
**5e**	100	92.4	85.7	74.4	69.3	62.9	854
**5f**	100	82.5	81.9	76.97	74.3	67.8	948
**5g**	100	96.7	95.1	90.2	85.8	79.2	*1193*
**5h**	100	94.97	81.7	78.7	72.8	66.2	900
**5i**	100	88.2	86.9	69.1	63.1	59.3	796
**5j**	100	84.2	66.1	61.5	39.1	38.5	196
**5k**	100	80.8	71.4	64.8	51.4	41.9	288
**5l**	100	70.5	60.6	57.2	48.1	39.5	223
**5m**	100	68.5	64.6	42.3	40.3	26.5	92
**5n**	100	54.3	51.5	50.8	46.4	40.5	130

**Table 5:** In vitro cytotoxic screening of **5a**–**n** against A549 human lung cancer cells

Comp.	Validity (%) for sample concentrations (µg/mL)									IC_50_ (µg/mL)
	0	1	10	31.25	62.50	125	250	500	1000	
**5a**	100	95.6	91.4	86.8	82.3	79.7	75.4	45.5	12.5	413
**5b**	100	91.2	85.4	83.99	67.8	64.3	57.1	44.2	17.8	402
**5c**	100	91.2	90.1	89.2	80.9	62.6	59.8	46.3	14.9	415
**5d**	100	98.4	91.7	88.9	82.2	78.8	73.01	58.6	18.1	574
**5e**	100	93.4	90.2	85.6	77.3	72.2	65.7	62.2	48.9	847
**5f**	100	94.97	89.8	86.96	83.8	79.2	75.4	65.5	12.6	567
**5g**	100	93.7	90.3	88.7	78.6	84.1	75.7	56.3	16.8	*572*
**5h**	100	92.9	91.4	90.3	88.7	85.7	82.7	77.2	68.0	1607
**5i**	100	93.3	92.2	91.4	85.6	81.3	76.9	63.2	49.3	896
**5j**	100	92.9	84.6	83.6	82.5	81.6	77.5	71.4	52.5	1046
**5k**	100	95.2	92.3	85.9	83.9	79.6	76.9	60.9	22.6	613
**5l**	100	96.5	93.6	75.4	72.2	68.01	62.9	51.9	41.3	577
**5m**	100	94.01	92.5	70.7	67.5	64.5	56.5	37.3	20.1	357
**5n**	100	94.2	91.2	83.1	78.6	70.7	58.9	27.3	8.02	380

**Table 6:** In vitro cytotoxic screening of **5a**–**n** against MDA-MB-231 human breast cancer cells

Comp.	Validity (%) for sample concentrations (µg/mL)								IC_50_ (µg/mL)
	0	1	10	31.25	62.5	125	250	500	
**5a**	100	98.3	96.7	93.6	86.1	85.9	82.5	75.8	1498
**5b**	100	97.8	97.4	90.8	88.6	87.3	83.6	81.5	1625
**5c**	100	97.1	95.7	95.8	92.1	86.3	84.5	83.04	1690
**5d**	100	96.8	94.4	93.7	92.4	88.4	87.2	85.7	1740
**5e**	100	97.3	96.3	93.6	92.6	85.5	75.9	73.7	1405
**5f**	100	98.1	97.2	88.95	84.7	81.03	79.6	77.5	1540
**5g**	100	92.6	87.3	80.9	72.2	67.7	63.2	58.5	*525*
**5h**	100	90.9	82.8	79.3	76.8	71.7	61.7	56.7	514
**5i**	100	96.00	83.8	61.00	56.2	52.2	50.5	46.1	334
**5j**	100	94.4	74.8	66.99	61.4	60.2	57.2	49.3	435
**5k**	100	92.3	85.8	68.7	60.8	56.7	50.6	42.98	330
**5l**	100	99.11	87.6	79.7	76.2	72.4	64.6	57.8	522
**5m**	100	97.3	89.5	85.4	88.3	76.9	65.6	50.6	505
**5n**	100	98.7	91.0	92.8	90.6	76.8	58.7	27.2	315

**Table 7:** In vitro cytotoxic screening of **6a**–**n** against WI-38 human normal fibroblast cells

Comp.	Validity (%) for sample concentrations (µg/mL)						IC_50_ (µg/mL)
	0	31.25	62.50	125	250	500	
**6a**	100	71.1	66.1	59.1	49.1	47.1	241
**6b**	100	94.1	87.9	71.6	62.6	57.7	520
**6c**	100	94.5	65.9	63.4	59.5	55.5	*515*
**6d**	100	92.01	88.1	78.6	67.5	67.7	*759*
**6e**	100	68.8	52.5	45.6	38.6	26.8	65.4
**6f**	100	49	44.8	35.6	32.3	30.4	29
**6g**	100	73.9	42.00	37.4	35.7	30.6	40
**6h**	100	85.01	70.6	60.4	51.8	49.4	487
**6i**	100	83.1	76.5	56.5	49.2	39.2	240
**6j**	100	87.3	81.4	66.3	60.9	37.2	356
**6k**	100	77.9	64.03	61.3	60.3	58.3	*528*
**6l**	100	94.01	76.4	70.2	52.5	39.02	350
**6m**	100	78.5	72.8	68.5	63.00	55.7	512
**6n**	100	98.6	85.9	53.2	43.3	39.8	212

**Table 8:** In vitro cytotoxic screening of **6a**–**n** against A549 human lung cancer cells

Comp.	Validity (%) for sample concentrations (µg/mL)									IC_50_ (µg/mL)
	0	1	10	31.25	62.50	125	250	500	1000	
**6a**	100	98.6	89.1	79.2	75.9	70.3	30.7	20.8	14.4	185
**6b**	100	95.7	86.9	85.8	83.5	77.8	68.2	35.2	36.3	338
**6c**	100	92.4	80.7	59.8	54.2	31.3	25.1	19.7	17.7	*85*
**6d**	100	97.9	92.6	77.2	67.3	49.02	24.6	20.4	19.4	*117*
**6e**	100	95.4	90.3	87.5	81.7	71.1	50.7	33.2	28.8	276
**6f**	100	91.3	89.8	86.2	83.2	67.3	46.6	38.6	25.4	230
**6g**	100	94.5	90.1	86.9	73.5	68.9	63.1	18.4	14.4	308
**6h**	100	89.5	80.9	78.98	75.8	63.5	37.9	33.03	30.3	192
**6i**	100	96.6	91.9	89.4	78.1	76.3	67.9	53.3	49.4	801
**6j**	100	95.6	87.8	76.6	68.2	54.00	31.3	29.2	28.3	175
**6k**	100	90.8	80.8	75.98	70.6	55.4	39.8	28.7	23.0	*203*
**6l**	100	85.04	72.2	58.1	53.6	43.1	34.0	30.3	27.9	95
**6m**	100	91.5	81.8	67.6	65.9	62.7	58.7	54.1	49.8	830
**6n**	100	93.3	83.6	67.9	64.2	52.7	43.2	39.1	36.3	159

**Table 9:** In vitro cytotoxic screening of **6a**–**n** against MDA-MB-231 human breast cancer cells

Comp.	Validity (%) for sample Concentrations (µg/mL)								IC_50_ (µg/mL)
	0	1	10	31.25	62.50	125	250	500	
**6a**	100	99.4	92.4	90.7	83.1	78.3	69.6	63.2	612
**6b**	100	95.9	93.4	89.7	84.2	79.8	72.9	68.9	749
**6c**	100	92.8	77.8	63.1	53.1	46.04	29.4	24.8	*104*
**6d**	100	93.3	89.1	75.8	63.3	53.1	27.7	24.00	*169*
**6e**	100	98.2	90.2	89.9	85.2	69.2	41.9	39.4	215
**6f**	100	91.8	89.6	83.00	72.4	59.7	37.2	28.9	188
**6g**	100	96.7	93.9	91.2	82.9	71.1	65.5	55.6	576
**6h**	100	94.3	90.4	85.8	77.8	73.6	68.5	65.8	685
**6i**	100	94.2	89.1	66.8	55.9	49.2	24.3	18.2	120
**6j**	100	96.3	92.9	75.6	73.1	69.01	53.2	50.6	508
**6k**	100	96.4	90.7	78.6	69.6	51.00	33.3	19.1	*131*
**6l**	100	96.9	90.4	85.5	71.9	69.8	66.7	60.9	603
**6m**	100	93.2	89.9	77.6	73.2	67.1	63.4	59.5	590
**6n**	100	96.2	89.5	86.8	81.3	73.2	67.1	59.2	584

Quantitatively, **5m**, **5n**, **5l**, **5d**, and **5k** showed moderate cytotoxic effect on normal cell lines with IC_50_ (92, 130, 223, 268, 288 µg/mL); respectively, as shown in Fig. 3 and Table 4 but less effective on A549 and MDA-MB-31 cancer cells. Briefly, **5m**, **5n**, **5b**, **5a**, **5c**, **5d**, and **5l** with IC_50_ (357, 380, 402, 413, 415, 574, and 577 µg/mL) on A549 cells as shown in Fig. 3 and Table 5; **5m**, **5n**, **5b**, **5a**, **5c**, **5f**, **5g**, **5d**, **5l**, and **5k** with (357, 380, 402, 413, 415, 567, 572, 574, 577, and 613 µg/mL) on MDA-MB-231 cells, respectively, as shown in Fig. 3 and Table 6. In conclusion, **5g** is the only compound that exerts a moderate anti-cancer activity on both lung and breast cancer cells.

Fluorescence-activated cell sorting (FACS) analysis for annexin V and PI staining to follow the mechanisms of cell death show that **5g** induce necrotic cell death as the following (15.8%, 16.7%, and 14.1% of total cell number) on WI-38, A549, and MDA-MB-231 cells, respectively. On the other hand, induce insignificant apoptotic cell death with (1.5%, 1.25%, and 1.34% of total cell number) as shown in Fig. 4.

Moreover, in vitro anti-proliferative effect of azetidinone derivatives **6a**–**n** on normal lung cells, lung and breast cancer cells and uncover the mechanisms of cell death in selected drugs which show anti-cancer activities.

Concisely, compounds **6e**, **6f**, and **6g** bearing 4-(dimethylamino)phenyl, 4-nitrophenyl and 4-carboxyphenyl moieties, respectively, exerted an observed cytotoxic activity with IC_50_ (65.4, 29, and 40 µg/mL); correspondingly, against WI-38 normal lung cells compared with taxol which induce cell death with IC_50_ (41 µg/mL) as shown in Fig. 5 and Table 7. In the case of A549 lung cancer cells, compounds **6a**, **6c**, **6d**, **6j**, **6k**, **6l**, and **6n** bearing phenyl, 4-cholorophenyl, 4-bromophenyl, furan-2-yl, thiophen-2-yl, 1*H*-pyrrol-2-yl, and quinolin-4-yl moieties, respectively, showed weak anti-proliferative activity with IC_50_ (185, 85, 117, 175, 203, 95, and 159 µg/mL); respectively, compared with taxol (IC_50_ 2.3 µg/mL) as shown in Fig. 5, Table 8. Moving to MDA-MB-231 breast cancer cells, the screening result showed that compounds **6c**, **6d**, **6f**, **6i**, and **6k** bearing 4-cholorophenyl, 4-bromophenyl, 4-nitrophenyl, styryl, and thiophen-2-yl moieties exhibited cytotoxicity with IC_50_ (104, 169, 188, 120, and 131 µg/mL); respectively, compared with (IC_50_ 40 µg/mL) for Taxol as illustrated in Fig. 5 and Table 9. In conclusion, we can quantitatively conclude that, compounds **6c**, **6d** and **6k** exerted ant-cancer activity on normal lung cells versus lung and breast cancer cells with IC_50_ (515, 759, and 528 µg/mL), (85, 117, and 203 µg/mL), and (104, 169, and 131 µg/mL), respectively.

Additional study using FACS analysis was done to expose the mechanism of cell death for compounds **6c**, **6d** and **6k**. Flow cytometry using annexin V and propidium Iodide show that, **6c**, **6d** and **6k** induced low necrotic cell death (14.5%, 14.1%, and 9.93%) of total cell number while inducing non-observed apoptotic cell death (1.22%, 1.34% and 0.61%) of total cell number as shown in Figs. 6, 7, 8, correspondingly. In the case of lung cancer cells, **6c**, **6d** and **6k** induced markedly apoptotic cell death with (27.32%, 36.3%, and 32.67%) while inducing insignificant necrotic cell death with (2.1%, 2%, and 1.71%) of total cell populations. More interestingly, the selective compounds show a highly significant apoptotic cell death induction with (80.32%, 55.355, and 67.25) of total cell number while inducing in visible necrotic cell death (2.15%, 6.515%, and 4.56%); respectively.

### Docking and molecular modeling study

Molecular Docking study of 28 new synthesized compounds **5a**–**n** and **6a**–**n** has been performed. The main idea was to build molecules that have the ability to intercalate between the DNA base pairs while in the same time be able to stabilize their intercalating complex through formation of different bonding with topoisomerase I amino acids. Molecular Docking study was done in order to comprehend the mechanism of interaction of the synthesized compounds with DNA topoisomerase I and to verify the difference in activity as antibacterial and anticancer between different synthesized analogues. Molecular Operating Environment (MOE^®^) version 2019.01, Chemical Computing Group (CCG) Inc., Montreal, Canada was used for this purpose [Molecular Operating Environment (MOE)], Version, Chemical Computing group Inc., Montreal, Quebec, Canada, 2016. http://www.chemcomp.com.].

**Fig. 9:**
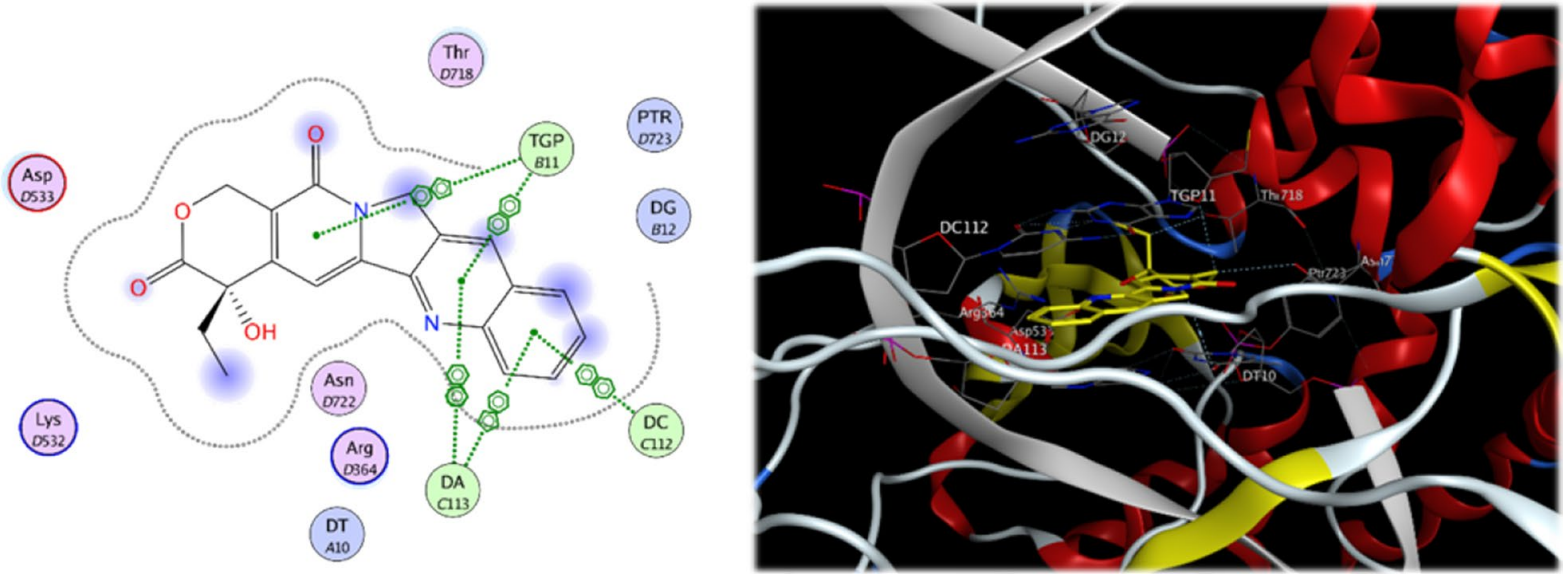
2D and 3D interaction of folate and DNA topoisomerase I enzyme

The crystal structure of DNA topoisomerase I was obtained from Protein Data Bank [https://www.rcsb.org] at 3.0°A resolution (PDB code: 1T8I). It consists of 592 amino acid residues in one chain. After preparation of the enzyme, molecular docking of the cocrystallised Camptothecin ligand was done (Fig. 9) with different placement protocol in order to choose the best methodology for docking. The Triangle matcher placement method showed RMSD value of less than 2 (1.3581) which indicates the confidence in the produced docking results. As can be seen from the 2D and 3D interaction between Camptothecin and DNA topoisomerase I enzyme, Camptothecin acts mainly through intercalation between DNA base pairs which halts the ability division of DNA double strand.

Molecular docking of the conformation database of the 42 synthesized compounds into the active site of DNA topoisomerase I was carried out using the mentioned protocol with the results refinement using force-field based scoring function GBVI/WSA dG which estimates the free energy of binding of the ligand from a given pose. The functional form is a sum of terms:

$$\Delta G \approx c + \alpha \left[ { \frac{2}{3}\left( {\Delta Ecoul + \Delta Esol} \right) + \Delta Evdw + \beta \Delta SAweighted} \right]$$

*C* is represents the average gain/loss of rotational and translational entropy. *α*, *β* is constants which were determined during training (along with *c*) and are forcefield-dependent. *Ecoul* is the columbic electrostatic term, which is calculated using currently loaded charges, using a constant dielectric of 1. *Esol* is the solvation electrostatic term which is calculated using the GB/VI solvation model. *Evdw* is the Van der Waals contribution to binding. *SAweighted* is the surface area weighted by exposure.

**Fig. 10:**
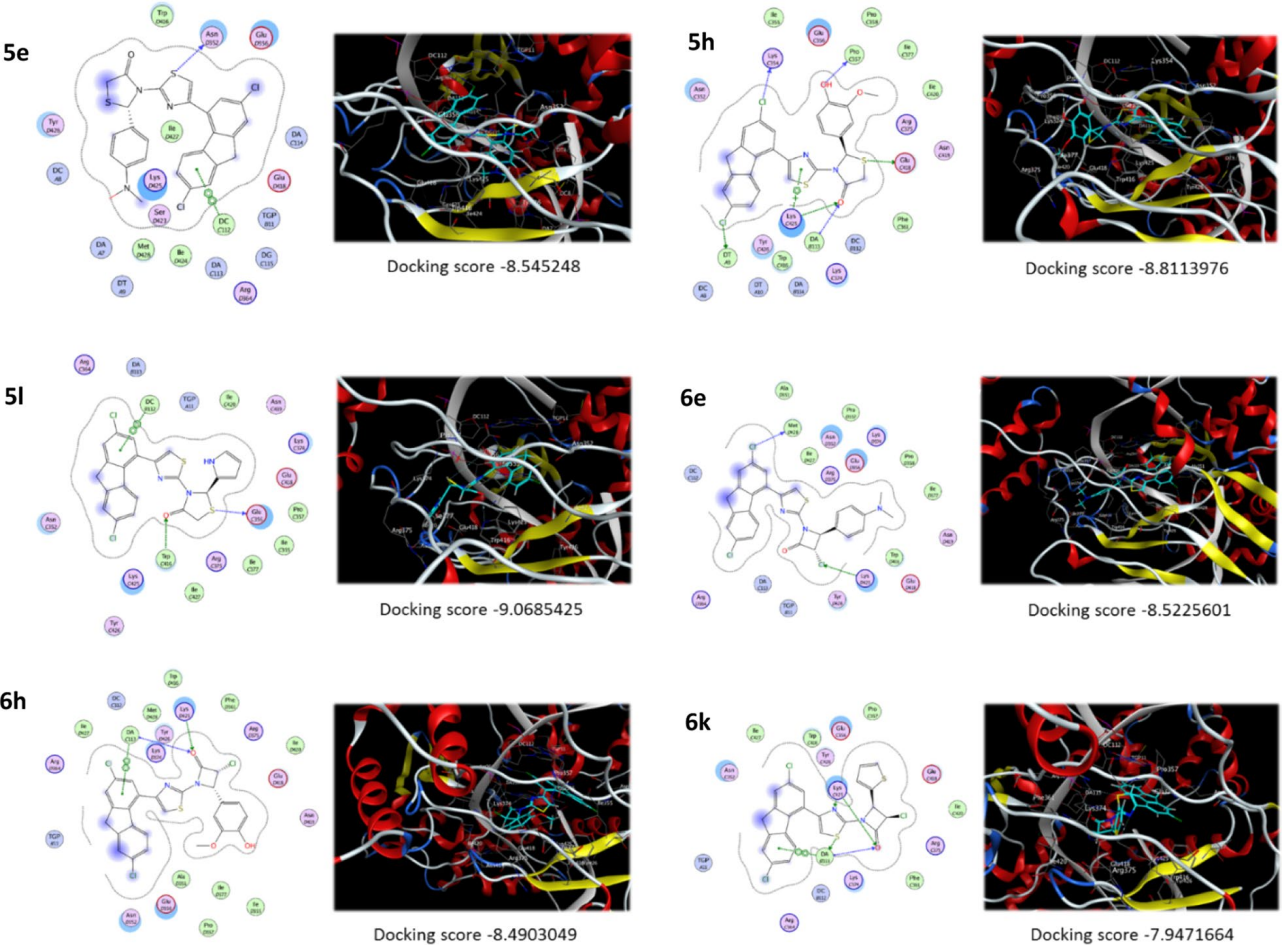
Molecular docking of compounds **5e**, **5h**, **5l**, **6e**, **6h**, and **6k**

The output docking results were arranged according to scoring function and explored using the browser function embedded in MOE software. Representation of 2D and 3D of the ligand interaction between all the synthesized compounds and DNA topoisomerase I enzymes is shown in Fig. 10. The synthesized compounds can be sorted into two different groups **5** and **6** according to the attachment to the (9*H*-fluoren-4-yl)thiazole; first the attachment is through thiazolino-4-one moiety and second the attachment is through β-lactam ring. Upon examining the scoring results, most of the highest active compounds showed better energy scores. So, compounds **5e**, **5h**, **5l**, **6e** and **6h** showed high scores in comparison with other analogues. The scores were in the range of − 9.0685 to − 8.4903 kcal/mole.

The details of the interactions are as the following: most of the compounds were able to intercalate between the DNA base pairs while forming hydrophobic interactions with the different nucleic acid skeleton and forming other types of interaction with the amino acid residues in the topoisomerase I enzyme. So, for all the compounds beside intercalation with DNA, the following binding interaction was present: Compound **5e** interacts with the active site through formation of hydrogen bond between the sulfur of the thiazole ring and ASN352 with a distance of 4.03 Ǻ; beside hydrophobic interactions with different amino acid residues like LYS425 and TYR426. Compound **5h** interacts with the active site through formation of 4 hydrogen bonds between the hydroxyl, chloro, carbonyl group and sulfur of the thiazolidine ring and PRO357, LYS354, LYS425 and GLU418, respectively. The distances of hydrogen bonds in order are 2.72, 2.92, 3.06 and 3.11 Ǻ. Compound **5l** interacts with the active site through formation of 2 hydrogen bonds between sulfur of thiazolidinone ring and carbonyl group on one side and GLU356 and TRP426 on the other hand with distances of 3.00 and 3.15 Ǻ, respectively. Compound **6e** interacts with the active site through formation of 2 hydrogen bonds between chloro groups on the fluorene moiety and on the β-lactam ring on one hand and MET428 and LYS425 on the other hand with distances of 3.76 and 3.72 Ǻ, respectively. Compound **6h** interacts with the active site through formation of 2 hydrogen bonds between keto group on the β-lactam ring and sulfur of the thiazole ring on one side and LYS374 and one of the DNA nucleic acids with a distance of 3.32 and 3.72 Ǻ, respectively. The high activity of group three (β-lactam) against both cancer cell lines and bacteria may be attributed to the opening of the β-lactam ring and the increase in the flexibility of the molecules.

## Conclusion

In this study, various 2,7-dichloro-9*H*-fluorene-based thiazolidinone and azetidinone derivatives were designed, synthesized, fully characterized and screened in vitro against various multidrug resistant microorganisms as well as against human lung carcinoma (A-549) and human breast carcinoma (MCF-7) cell lines. The results indicated that 2,7-dichloro-9*H*-fluorene-based azetidinones are more efficacious antimicrobial and anticancer agents compared to dichloro-9*H*-fluorene-based thiazolidinones analogues. Hence, there is adequate scope for further study in developing such compounds as a good lead activity.

## Experimental

### Chemistry

#### General methods

All Chemicals and solvents used purchased from Sigma-Aldrich are spectroscopic grade and used without further purifications. Melting points were determined on a Stuart SMP3 melting point apparatus and are uncorrected. FT-IR spectra were recorded on a Shimadzu IR-3600 FT-IR spectrometer in KBr pellets. NMR spectra were acquired on a Bruker Avance 400 instrument (400 MHz for ^1^H, 100 MHz for ^13^C) in DMSO-*d*_*6*_ solutions, using residual solvent signals as internal standards. Starting materials 2,7-dichloro-9*H*-fluorene (**2**) and 2-chloro-1-(2,7-dichloro-9*H*-fluoren-4-yl)ethanone (**3**) were prepared according to our previously reported method [31].

##### Synthesis of 4-(2,7-dichloro-9*H*-fluoren-4-yl)thiazol-2-amine (3)

A mixture of chloroacetyl derivative **2** (15.55 g, 50 mmol) and thiourea (5.70 g, 75 mmol) in ethanol (250 mL) was refluxed for 3 h. The reaction mixture was cooled and neutralized with saturated aqueous solution of sodium biocarbonate. The obtained solid product was filtered off, washed with cold water (3 × 50 mL), then with cold ethanol (3 × 10 mL), dried and recrystallized from ethanol to afford 16.15 gm (97%) of pure 2-aminothiazole derivative **3** as pale yellow crystals, m.p. 199–200 °C. FT-IR (KBr): *ν* (cm^−1^) 3282, 3106 (NH_2_), 1639 (C=N); ^1^H-NMR (DMSO-*d*_*6*_): δ 7.66 (s, 1H, Flu-H), 7.63 (s, 1H, Flu-H), 7.55 (d, 1H, *J* = 5.5 Hz, Flu-H), 7.37 (s, 1H, Flu-H), 7.31 (d, 1H, *J* = 5.5 Hz, Flu-H), 7.18 (s, 2H, NH_2_), 6.77 (s, 1H, Thiazolyl-H), 4.00 (s, 2H, CH_2_); ^13^C-NMR (DMSO-*d*_*6*_): δ 168.8 (C=N), 148.9 (C), 146.7 (C), 146.3 (C), 139.1 (C), 136.9 (C), 133.5 (C), 131.9 (C), 131.2 (C), 128.7 (CH), 126.9 (CH), 125.3 (CH), 125.1 (CH), 124.9 (CH), 105.5 (Thiazole-CH), 36.7 (CH_2_).

##### Synthesis of 4-(2,7-dichloro-9*H*-fluoren-4-yl)-*N*-(aryl/heteroaryl-methylene)thiazol-2-amine **4a**–**n**

A mixture of **3** (10 mmol, 3.33 g) and appropriate aromatic aldehyde (10 mmol) in absolute ethanol (50 mL) was heated under reflux for 4 h in the presence of two drops of dry piperidine. The reaction was concentrated and left to cool. The solid products were filtered and recrystallized from ethanol to give compounds **4a**–**n** in 71–96% yields.

##### Synthesis of 2-(aryl/heteroaryl)-3-(4-(2,7-dichloro-9*H*-fluoren-4-yl)thiazol-2-yl)thiazolidin-4-ones (**5a**–**n**)

A mixture of Schiff base 4 (1.0 mmol) and thioglycolic acid (1.5 mmol) was stirred in THF with ice cooling for 5 min, followed by addition of DCC (308 mg, 1.5 mmol) was added to the reaction mixture at 0 °C, and the reaction mixture was stirred for an additional 50 min at room temperature. Dicyclohexylurea was filtered off and the filtrate was concentrated under reduced pressure. The solid product was collected, washed thoroughly with diluted sodium bicarbonate solution, dried and recrystallized from methanol to afford the thiazolidinone derivatives **5a**–**n**.

###### 3-(4-(2,7-Dichloro-9*H*-fluoren-4-yl)thiazol-2-yl)-2-phenylthiazolidin-4-one (**5a**)

Pale yellow crystals, yield (76%), m.p. 79–82 °C; FT-IR (KBr): ν (cm^−1^) 3064 (CH arom.), 2920 (CH aliph.), 1700 (C=O), 1621 (C=N); ^1^H NMR (400 MHz, DMSO-*d*_6_): δ 7.66 (s, 1H, Flu-H), 7.60 (s, 1H, Flu-H), 7.48 (d, *J* = 8.0 Hz, 1H, Flu-H), 7.38 (d, *J* = 8.0 Hz, 2H, Ph-H), 7.28 (s, 1H, Flu-H), 7.20–7.17 (m, 2H, Ph-H), 6.99 (d, *J* = 8.0 Hz, 1H, Flu-H), 6.98–6.95 (m, 1H, Ph-H), 6.93 (s, 1H, Thiazole-H), 6.76 (s, 1H, CH), 3.98 (s, 2H, CH_2_), 3.60 (s, 2H, CH_2_); ^13^C NMR (100 MHz, DMSO-*d*_6_): δ 171.5 (C=O), 166.5 (C=N), 148.8 (C), 146.7 (C), 146.1 (C), 140.1 (C), 138.9 (C), 133.3 (C), 133.1 (C), 131.8 (C), 131.5 (C), 129.9 (CH), 129.7 (CH), 129.1 (CH), 128.9 (CH), 128.4 (CH), 127.1 (CH), 126.9 (CH), 125.4 (CH), 106.9 (Thiazole-CH), 66.2 (CH), 37.0 (CH_2_), 35.7 (CH_2_).

###### 3-(4-(2,7-Dichloro-9*H*-fluoren-4-yl)thiazol-2-yl)-2-(4-methoxyphenyl)thiazolidin-4-one (**5b**)

Pale yellow crystals, yield (80%), m.p. 74–76 °C; FT-IR (KBr): ν (cm^−1^) 3008 (CH arom.), 2930 (CH aliph.), 1694 (C=O), 1600 (C=N); ^1^H NMR (400 MHz, DMSO-*d*_6_): δ 7.87 (d, *J* = 8.0 Hz, 1H, Flu-H), 7.67 (d, *J* = 8.0 Hz, 2H, Ph-H), 7.57 (s, 1H, Flu-H), 7.48 (s, 1H, Flu-H), 7.45 (d, *J* = 8.0 Hz, 1H, Flu-H), 7.32 (s, 1H, Flu-H), 7.11 (s, 1H, Thiazole-H), 6.93 (d, *J* = 8.0 Hz, 2H, Ph-H), 6.83 (s, 1H, CH), 4.00 (s, 2H, CH_2_), 3.87 (s, 3H, CH_3_), 3.76 (s, 2H, CH_2_); ^13^C NMR (100 MHz, DMSO- *d*_6_): δ 173.3 (C=O), 168.9 (C=N), 148.7 (C), 146.6 (C), 146.1 (C), 139.2 (C), 136.9 (C), 133.9 (C), 132.8 (C), 132.2 (C), 131.9 (C), 131.5 (C), 129.2 (CH), 128.6 (CH), 126.9 (CH), 125.4 (CH), 125.2 (CH), 124.9 (CH), 123.3 (CH), 105.7 (Thiazole-CH), 82.4 (CH), 48.4 (CH_3_) 35.9 (CH_2_), 32.9 (CH_2_).

###### 2-(4-Chlorophenyl)-3-(4-(2,7-dichloro-9*H*-fluoren-4-yl)thiazol-2-yl)thiazolidin-4-one (**5c**)

Pale yellow crystals, yield (78%), m.p. 83–85 °C; FT-IR (KBr): ν (cm^−1^) 3025 (CH arom.), 2928 (CH aliph.), 1775 (C=O), 1694 (C=N); ^1^H NMR (400 MHz, DMSO-*d*_6_): δ 7.64 (d, *J* = 8.0 Hz, 2H, Ph-H), 7.57 (s, 1H, Flu-H), 7.52 (d, *J* = 8.0 Hz, 1H, Flu-H), 7.44 (s, 1H, Flu-H), 7.32 (s, 1H, Flu-H), 7.18 (d, *J* = 8.0 Hz, 1H, Flu-H), 6.95 (s, 1H, Thiazole-H), 6.76 (d, *J* = 8.0 Hz, 2H, Ph-H), 6.53 (s, 1H, CH), 3.95 (s, 2H, CH_2_), 3.32 (s, 2H, CH_2_); ^13^C NMR (100 MHz, DMSO-*d*_6_): δ 173.3 (C=O), 167.0 (C=N), 156.5 (C), 148.8 (C), 147.8 (C), 146.6 (C), 145.9 (C), 140.6 (C), 138.4 (C), 133.2 (C), 132.8 (C), 132.2 (C), 129.3 (CH), 129.1 (CH), 128.9 (CH), 127.7 (CH), 126.8 (CH), 123.8 (CH), 114.3 (CH), 105.7 (Thiazole-CH), 66.2 (CH), 36.8 (CH_2_), 33.5 (CH_2_).

###### 2-(4-Bromophenyl)-3-(4-(2,7-dichloro-9*H*-fluoren-4-yl)thiazol-2-yl)thiazolidin-4-one (**5d**)

Orange crystals, yield (73%), m.p. 155–157 °C; FT-IR (KBr): ν (cm^−1^) 3018 (CH arom.), 2927 (CH aliph), 1776 (C=O), 1636 (C=N); ^1^H NMR (400 MHz, DMSO-*d*_6_): δ 7.63 (s, 1H, Flu-H), 7.52 (d, *J* = 8.0 Hz, 2H, Flu-H), 7.24 (s, 1H, Flu-H), 7.18 (s, 1H, Flu-H), 7.08 (d, *J* = 8.0 Hz, 2H, Ph-H), 6.78 (s, 1H, Thiazole-H), 6.66 (s, 1H, CH), 6.56 (d, *J* = 8.0 Hz, 2H, Ph-H), 3.96 (s, 2H, CH_2_), 3.59 (s, 2H, CH_2_); ^13^C NMR (100 MHz, DMSO-*d*_6_): δ 171.6 (C=O), 168.0 (C=N), 156.6 (C), 150.4 (C), 147.9 (C), 146.6 (C), 145.8 (C), 138.4 (C), 137.1 (C), 132.1 (C), 131.7 (C), 131.5 (C), 129.3 (CH), 128.1 (CH), 126.8 (CH), 125.5 (CH), 125.2 (CH), 124.1 (CH), 114.2 (CH), 112.4 (CH), 111.5 (Thiazole-CH), 68.1 (CH), 36.6 (CH_2_), 34.4 (CH_2_).

###### 3-(4-(2,7-Dichloro-9*H*-fluoren-4-yl)thiazol-2-yl)-2-(4-(dimethylamino)phenyl)thiazolidin-4-one (**5e**)

Pale yellow crystals, yield (84%), m.p. 94–96 °C; FT-IR (KBr): ν (cm^−1^) 3074 (CH arom.), 2926 (CH aliph.), 1689 (C=O), 1607 (C=N); ^1^H NMR (400 MHz, DMSO-*d*_6_): δ 8.23 (d, *J* = 8.0 Hz, 2H, Ph-H), 7.72 (d, *J* = 8.0 Hz, 1H, Flu-H), 7.64 (s, 1H, Flu-H), 7.56 (s, 1H, Flu-H), 7.33 (s, 1H, Flu-H), 7.16 (d, *J* = 8.0 Hz, 1H, Flu-H), 6.96 (s, 1H, Thiazole-H), 6.89 (d, *J* = 8.0 Hz, 2H, Ph-H), 6.35 (s, 1H, CH), 3.95 (s, 2H,CH_2_), 3.42 (s, 2H, CH_2_), 1.74 (s, 6H, 2CH_3_); ^13^C NMR (100 MHz, DMSO-*d*_6_): δ 171.3 (C=O), 166.8 (C=N), 148.8 (C), 147.7 (C), 147.38 (C), 146.6 (C), 146.0 (C), 138.8 (C), 137.1 (C), 132.9 (C), 131.8 (C), 131.5 (C), 128.6 (CH), 128.4 (CH), 127.9 (CH), 126.6 (CH), 125.2 (CH), 124.5 (CH), 124.2 (CH), 107.4 (Thiazole-CH), 68.8 (CH), 61.3 (CH_3_), 36.9 (CH_2_), 33.8 (CH_2_).

###### 3-(4-(2,7-Dichloro-9*H*-fluoren-4-yl)thiazol-2-yl)-2-(4-nitrophenyl)thiazolidin-4-one (**5f**)

Yellow crystals, yield (76%), m.p. 90–92 °C; FT-IR (KBr): ν (cm^−1^) 3074 (CH arom.), 2930 (CH aliph.), 1707 (C=O), 1600 (C=N); ^1^H NMR (400 MHz, DMSO-*d*_6_): δ 7.76 (d, *J* = 8.0 Hz, 1H, Flu-H), 7.67 (s, 1H, Flu-H), 7.58 (s, 1H, Flu-H), 7.35 (s, 1H, Flu-H), 7.24 (d, *J* = 8.0 Hz, 2H, Ph-H), 7.18 (s, 1H, Flu-H), 7.10 (d, *J* = 8.0 Hz, 2H, Ph-H), 6.86 (s, 1H, Thiazole-H), 6.68 (s, 1H, CH), 3.96 (s, 2H,CH_2_), 3.60 (s, 2H,CH_2_); ^13^C NMR (100 MHz, DMSO-*d*_6_): δ 168.9 (C=O), 166.8 (C=N), 148.7 (C), 146.6 (C), 146.2 (C), 139.2 (C), 136.9 (C), 133.6 (C), 132.7 (C), 132.5 (C), 131.9 (C), 131.5 (C), 128.5 (CH), 127.4 (CH), 127.4 (CH), 126.9 (CH), 125.4 (CH), 125.1 (CH), 124.9 (CH), 105.7 (Thiazole-CH), 102.6 (CH), 36.9 (CH_2_), 33.6 (CH_2_).

###### 4-(3-(4-(2,7-Dichloro-9*H*-fluoren-4-yl)thiazol-2-yl)-4-oxothiazolidin-2-yl)benzoic acid (**5g**)

Yellow crystals, yield (90%), m.p. 103–105 °C; FT-IR (KBr): ν (cm^−1^) 3326 (OH), 3065 (CH arom.), 2930 (CH aliph.), 1778 (C=O), 1696 (C=O), 1628 (C=N); ^1^H NMR (400 MHz, DMSO-*d*_6_): δ 9.10 (m, 1H, OH), 7.64–7.58 (m, 3H, Ph-H & Flu-H), 7.54 (s, 1H, Flu-H), 7.42 (d, *J* = 8.0 Hz, 1H, Flu-H), 7.38 (d, *J* = 8.0 Hz, 1H, Flu-H), 7.24 (s, 1H, Flu-H), 6.91–6.83 (m, 3H, Ph-H & Thiazole-H), 6.68 (s, 1H, CH), 3.92 (s, 2H, CH_2_), 3.69 (s, 2H, CH_2_); ^13^C NMR (100 MHz, DMSO-*d*_6_): δ 191.4 (C=O), 171.6 (C=O), 157.1 (C=N), 156.6 (C), 147.9 (C), 147.7 (C), 147.2 (C), 146.7 (C), 146.3 (C), 138.5 (C), 136.8 (C), 132.1 (C), 131.5 (C), 129.2 (CH), 126.8 (CH), 125.5 (CH), 125.3 (CH), 124.3 (CH), 117.9 (CH), 115.7 (CH), 114.3 (CH), 111.2 (Thiazole-CH), 68.1 (CH), 36.8 (CH_2_), 34.7 (CH_2_).

###### 3-(4-(2,7-Dichloro-9*H*-fluoren-4-yl)thiazol-2-yl)-2-(4-hydroxy-3-methoxyphenyl)thiazolidin-4-one (**5h**)

Yellow crystals, yield (76%), m.p. 85–87 °C; FT-IR (KBr): ν (cm^−1^) 3328 (OH), 3069 (CH arom.), 2930 (CH aliph.), 1689 (C=O), 1600 (C=N); ^1^H NMR (400 MHz, DMSO-*d*_6_): δ 7.66–7.61 (m, 3H, Flu-H & Ph-H), 7.51 (d, *J* = 8.0 Hz, 1H, Flu-H), 7.35 (s, 1H, Flu-H), 7.31 (d, *J* = 8.0 Hz, 1H, Flu-H), 7.16 (s, 1H, Flu-H), 7.01–6.99 (m, 2H, Ph-H & Thiazole-H), 6.77 (s, 1H, CH), 4.38 (s, 2H, CH_2_), 4.03 (s, 2H, CH_2_), 3.98 (s, 3H, CH_3_); ^13^C NMR (100 MHz, DMSO-*d*_6_) δ 168.9 (C=O), 157.1 (C=N), 148.7 (C), 147.7 (C), 146.63 (C), 146.2 (C), 146.0 (C), 139.2 (C), 137.2 (C), 136.9 (C), 133.6 (C), 131.9 (C), 131.5 (C), 129.0 (CH), 128.5 (CH), 127.0 (CH), 126.9 (CH), 125.4 (CH), 125.2 (CH), 124.9 (CH), 105.7 (Thiazole-CH), 62.2 (CH), 47.9 (CH_3_), 37.5 (CH_2_), 33.8 (CH_2_).

###### 3-(4-(2,7-Dichloro-9*H*-fluoren-4-yl)thiazol-2-yl)-2-styrylthiazolidin-4-one (**5i**)

Pale yellow crystals, yield (72%), m.p. 114–116 °C; FT-IR (KBr): ν (cm^−1^): 3096 (CH arom.), 2930 (CH aliph.), 1689 (C=O), 1625 (C=N); ^1^H NMR (400 MHz, DMSO-*d*_6_): δ 7.66 (s, 1H, Flu-H), 7.60–7.53 (m, 2H, Flu-H), 7.49 (d, *J* = 4.0 Hz, 1H, Flu-H), 7.34 (s, 1H, Flu-H), 7.30–7.27 (m, 3H, Ph-H), 7.19–7.17 (m, 3H, Ph-H & Thiazole-H), 6.83–6.75 (m, 2H, CH=CH), 6.32 (dd, *J* = 8.0, 4.0 Hz, 1H, CH), 3.98 (s, 2H, CH_2_), 3.45 (dd, *J* = 12.0, 4.0 Hz, 2H, CH_2_); ^13^C NMR (100 MHz, DMSO-*d*_6_): δ 168.9 (C=O), 166.2 (C=N), 153.7 (C), 149.5 (C), 148.7 (C), 147.0 (C), 146.1 (C), 137.0 (C), 135.5 (C), 133.0 (C), 131.5 (C), 129.5 (CH), 129.2 (CH), 128.7 (CH), 127.7 (CH), 127.1 (CH), 126.9 (CH), 125.7 (CH), 125.4 (CH), 124.5 (CH), 118.4 (CH), 105.7 (Thiazole-CH), 61.8 (CH), 36.9 (CH_2_), 33.8 (CH_2_).

###### 3-(4-(2,7-Dichloro-9H-fluoren-4-yl)thiazol-2-yl)-2-(furan-2-yl)thiazolidin-4-one (**5j**)

Pale yellow crystals, yield (64%), m.p. 139–141 °C; FT-IR (KBr): ν (cm^−1^) 3099 (CH arom.), 2927 (CH aliph.), 1689 (C=O), 1636 (C=N); ^1^H NMR (400 MHz, DMSO-*d*_6_): δ 7.88 (d, *J* = 8.0 Hz, 1H, Flu-H), 7.62 (d, *J* = 8.0 Hz, 1H, Furyl-H), 7.51 (d, *J* = 8.0 Hz, 1H, Flu-H), 7.45 (s, 1H, Flu-H), 7.41 (d, *J* = 8.0 Hz, 1H, Furyl-H), 7.35 (s, 1H, Flu-H), 7.31 (s, 1H, Flu-H), 7.29–7.17 (m, 2H, Furyl-H & Thiazole-H), 4.06 (s, 2H, CH_2_), 3.92 (s, 2H, CH_2_); ^13^C NMR (100 MHz, DMSO-*d*_6_): δ 172.6 (C=O), 168.9 (C=N), 155.3 (C), 148.7 (C), 146.6 (C), 146.1 (C), 139.2 (C), 136.9 (C), 133.5 (C), 131.9 (C), 131.5 (C), 128.5 (CH), 126.9 (CH), 125.3 (CH), 125.1 (CH), 124.9 (CH), 122.9 (CH), 121.7 (CH), 113.2 (CH), 105.7 (Thiazole-CH), 69.0 (CH), 36.9 (CH_2_), 33.8 (CH_2_).

###### 3-(4-(2,7-Dichloro-9*H*-fluoren-4-yl)thiazol-2-yl)-2-(thiophen-2-yl)thiazolidin-4-one (**5k**)

Pale yellow crystals, yield (67%), m.p. 165–167 °C; FT-IR (KBr): ν (cm^−1^) 3029 (CH arom.), 2926 (CH aliph.), 1688 (C=O), 1636 (C=N); ^1^H NMR (400 MHz, DMSO-*d*_6_): δ 7.62 (d, *J* = 4.0 Hz, 1H, Flu-H), 7.51 (d, *J* = 8.0 Hz, 1H, Thienyl-H), 7.35 (d, *J* = 4.0 Hz, 1H, Flu-H), 7.31–7.29 (m, 2H, Flu-H & Thienyl-H), 7.22 (s, 1H, Flu-H), 7.17 (s, 1H, Flu-H), 7.00–6.98 (s, 1H, Thiazole-H), 6.90–6.87 (m, 1H, Thienyl-H), 6.76 (s, 1H, CH), 4.01 (s, 2H, CH_2_), 3.93 (dd, *J* = 8.0, 4.0 Hz, 2H, CH_2_); ^13^C NMR (100 MHz, DMSO-*d*_6_): δ 168.9 (C=O), 166.0 (C=N), 148.6 (C), 146.6 (C), 146.1 (C), 143.5 (C), 139.1 (C), 136.9 (C), 133.5 (C), 131.9 (C), 131.5 (C), 128.9 (CH), 128.5 (CH), 126.9 (CH), 125.8 (CH), 125.4 (CH), 125.1 (CH), 124.9 (CH), 119.6 (CH), 105.7 (Thiazole-CH), 62.0 (CH), 44.4 (CH_2_), 36.9 (CH_2_).

###### 3-(4-(2,7-Dichloro-9*H*-fluoren-4-yl)thiazol-2-yl)-2-(1*H*-pyrrol-2-yl)thiazolidin-4-one (**5l**)

Yellow crystals, yield (75%), m.p. 124–126 °C; FT-IR (KBr): ν (cm^−1^) 3459 (NH), 3099 (CH arom.), 2926 (CH aliph.), 1680 (C=O), 1636 (C=N); ^1^H NMR (400 MHz, DMSO-*d*_6_): δ 7.81 (d, *J* = 8.0 Hz, 1H, Flu-H), 7.61 (d, *J* = 8.0 Hz, 1H, Flu-H), 7.52 (s, 1H, Flu-H), 7.43 (s, 1H, Flu-H), 7.34–7.28 (m, 2H, Thiazole-H & Pyrrole-H), 7.18 (s, 1H, Flu-H), 6.97 (d, *J* = 8.0 Hz, 1H, Pyrrole-H), 6.76–7.64 (m, 2H, Pyrrole-H & CH), 6.07 (s, 1H, NH), 4.00–3.90 (m, 4H, 2CH_2_); ^13^C NMR (100 MHz, DMSO-*d*_6_): δ 171.9 (C=O), 168.9 (C=N), 150.6 (C), 149.9 (C), 148.7 (C), 146.7 (C), 146.1 (C), 136.9 (C), 133.5 (C), 131.9 (C), 131.5 (C), 128.5 (CH), 126.9 (CH), 126.7 (CH), 125.3 (CH), 124.9 (CH), 122.1 (CH), 121.3 (CH), 120.6 (CH), 105.6 (Thiazole-CH), 66.9 (CH), 36.9 (CH_2_), 34.4 (CH_2_).

###### 3-(4-(2,7-Dichloro-9*H*-fluoren-4-yl)thiazol-2-yl)-2-(pyridin-4-yl)thiazolidin-4-one (**5m**)

Orange crystals, yield (72%), m.p. 110–112 °C; FT-IR (KBr): ν (cm^−1^) 3097 (CH arom.), 2928 (CH aliph.), 1702 (C=O), 1636 (C=N), 1600 (C=N); ^1^H NMR (400 MHz, DMSO-*d*_6_): δ 8.86 (d, *J* = 8.0 Hz, 2H, Py-H), 8.07 (s, 1H, Flu-H), 7.71 (d, *J* = 8.0 Hz, 1H, Flu-H), 7.37 (d, *J* = 8.0 Hz, 1H, Flu-H), 7.26 (s, 2H, Flu-H), 6.96 (s, 1H, Thiazole-H), 6.70 (d, *J* = 8.0 Hz, 2H, Py-H), 6.18 (s, 1H, CH), 4.11 (s, 2H, CH_2_), 3.59 (s, 2H, CH_2_); ^13^C NMR (100 MHz, DMSO-*d*_6_): δ 168.7 (C=O), 166.5 (C=N), 153.9 (C), 150.8 (C), 143.9 (C), 143.4 (C), 134.0 (C), 131.4 (C), 130.9 (C), 130.2 (C), 130.0 (C), 128.2 (CH), 127.8 (CH), 127.5 (CH), 126.8 (CH), 126.2 (CH), 124.8 (CH), 124.5 (CH), 112.7 (CH), 106.3 (Thiazole-CH), 65.7 (CH), 36.9 (CH_2_), 34.4 (CH_2_).

###### 3-(4-(2,7-Dichloro-9*H*-fluoren-4-yl)thiazol-2-yl)-2-(quinolin-4-yl)thiazolidin-4-one (**5n**)

Pale yellow crystals, yield (90%), m.p. 204–206 °C; FT-IR (KBr): ν (cm^−1^) 3099 (CH arom.), 2924 (CH aliph.), 1636 (C=O), 1583 (C=N), 1538 (C=N); ^1^H NMR (400 MHz, DMSO-*d*_6_): δ 7.90–7.87 (m, 2H, Quinoline-H), 7.81–7.79 (m, 3H, Flu-H & Quinoline-H), 7.73–7.71 (m, 2H, Flu-H), 7.37–7.34 (m, 2H, Flu-H & Quinoline-H), 7.26–7.23 (m, 2H, Flu-H & Quinoline-H), 7.07 (s, 1H, Thiazole-H), 7.00 (s, 1H, CH), 4.34 (s, 2H, CH_2_), 3.61 (s, 2H, CH_2_); ^13^C NMR (100 MHz, DMSO-*d*_6_): δ 168.7 (C=O), 165.9 (C=N), 160.1 (CH=N), 154.3 (C), 153.9 (C), 152.65 (C), 150.28 (C), 143.79 (C), 143.3 (C), 139.3 (C), 136.9 (C), 135.4 (C), 132.9 (C), 129.6 (CH), 129.4 (CH), 129.13 (CH), 128.91 (CH), 128.65 (CH), 127.63 (CH), 125.99 (CH), 120.2 (CH), 119.5 (CH), 113.3 (CH), 101.9 (Thiazole-CH), 65.9 (CH), 36.9 (CH_2_), 34.2 (CH_2_).

##### Synthesis of 3-chloro-4-(aryl/heteroaryl)-1-(4-(2,7-dichloro-9*H*-fluoren-4-yl)thiazol-2-yl)azetidin-2-ones **6a**–**n**

To Schiff s base **4a**–**n** (1 mmol) in dry DMF (10 mL), chloroacetyl chloride (1.2 mmol) was added with stirring at room temperature during 15 min. The mixture was further stirred at room temperature for 5 h. The mixture was poured onto crushed ice. The obtained product was filtered, washed with water and recrystallized from ethanol to get pure azetidinone derivatives **6a**–**n**.

###### 3-Chloro-1-(4-(2,7-dichloro-9*H*-fluoren-4-yl)thiazol-2-yl)-4-phenylazetidin-2-one (**6a**)

Yellow crystals, yield (68%), m.p. 118–120 °C; FT-IR (KBr): ν (cm^−1^) 3062 (CH arom.), 2927 (CH aliph.), 1695 (C=O), 1650 (C=N); ^1^H NMR (400 MHz, DMSO-*d*_6_): δ 7.96 (s, 1H, Flu-H), 7.90 (d, *J* = 8.0 Hz, 1H, Flu-H), 7.84 (s, 1H, Flu-H), 7.78 (s, 1H, Flu-H), 7.72–7.65 (m, 2H, Ph-H), 7.54–7.51 (m, 2H, Ph-H), 7.41 (d, *J* = 8.0 Hz, 1H, Flu-H), 7.29–7.26 (m, 1H, Ph-H), 7.22 (s, 1H, Thiazole-H), 7.14 (d, *J* = 12.0 Hz, 1H, CH–N), 4.45 (d, *J* = 12.0 Hz, 1H, CH–Cl), 4.00 (s, 2H, CH_2_); ^13^C NMR (100 MHz, DMSO-*d*_6_): δ 169.1 (C=O), 165.8 (C=N), 158.0 (C), 147.8 (C), 146.7 (C), 146.2 (C), 138.8 (C), 137.0 (C), 132.4 (C), 132.1 (C), 131.7 (C), 129.6 (CH), 128.7 (CH), 127.5 (CH), 126.6 (CH), 125.5 (CH), 124.6 (CH), 122.9 (CH), 113.3 (CH), 107.0 (Thiazole-CH), 69.0 (CH–N), 57.3 (CH–Cl), 36.9 (CH_2_).

###### 3-Chloro-1-(4-(2,7-dichloro-9*H*-fluoren-4-yl)thiazol-2-yl)-4-(4-methoxyphenyl)azetidin-2-one (**6b**)

Yellow crystals, yield (98%), m.p. 110–112 °C; FT-IR (KBr): ν (cm^−1^) 3062 (CH arom.), 2951 (CH aliph.), 1691 (C=O), 1598 (C=N); ^1^H NMR (400 MHz, DMSO-*d*_6_): δ 7.87 (d, *J* = 5.0 Hz, 1H, Flu-H), 7.70 (s, 1H, Flu-H), 7.64 (d, *J* = 8.0 Hz, 2H, Ph-H), 7.50 (s, 1H, Flu-H), 7.40 (s, 1H, Flu-H), 7.28–7.26 (m, 3H, Flu-H & Ph-H), 7.21 (s, 1H, Thiazole-H), 7.13 (d, *J* = 8.0 Hz, 1H, CH–N), 4.45 (s, 3H, CH_3_), 4.21 (d, *J* = 8.0 Hz, 1H, CH–Cl), 4.01 (s, 2H, CH_2_); ^13^C NMR (100 MHz, DMSO-*d*_6_): δ 169.1 (C=O), 165.7 (C=N), 158.0 (C), 147.9 (C), 146.8 (C), 146.2 (C), 138.8 (C), 137.0 (C), 132.3 (CH), 131.6 (C), 130.1 (C), 128.9 (CH), 127.1 (CH), 125.9 (CH), 125.5 (CH), 124.6 (CH), 123.4 (C), 122.1 (C), 114.9 (CH), 113.30 (Thiazole-CH), 69.0 (CH–N), 57.3 (CH–Cl), 40.1 (CH_3_), 36.9 (CH_2_).

###### 3-Chloro-4-(4-chlorophenyl)-1-(4-(2,7-dichloro-9*H*-fluoren-4-yl)thiazol-2-yl)azetidin-2-one (**6c**)

Yellow crystals, yield (96%), m.p. 105–106 °C; FT-IR (KBr): ν (cm^−1^) 3063 (CH arom.), 2929 (CH aliph.), 1697 (C=O), 1593 (C=N); ^1^H NMR (400 MHz, DMSO-*d*_6_) δ 7.95 (d, *J* = 5.0 Hz, 1H, Flu-H), 7.72 (s, 1H, Flu-H), 7.66 (s, 1H, Flu-H), 7.51 (d, *J* = 8.0 Hz, 2H, Ph-H), 7.40 (m, 3H, Flu-H & Ph-H), 7.28 (s, 1H, Flu-H), 7.21 (s, 1H, Thiazole-H), 7.12 (d, *J* = 8.0 Hz, 1H, CH–N), 4.46 (d, *J* = 8.0 Hz, 1H, CH–Cl), 4.03 (s, 2H, CH_2_); ^13^C NMR (100 MHz, DMSO-*d*_6_): δ 169.1 (C=O), 165.8 (C=N), 158.0 (C), 147.8 (C), 146.8 (C), 146.3 (C), 138.8 (C), 137.04 (C), 132.5 (C), 132.1 (C), 131.6 (C), 129.2 (CH), 129.0 (C), 127.7 (CH), 127.1 (CH), 126.0 (CH), 125.6 (CH), 124.6 (CH), 114.9 (CH), 105.4 (Thiazole-CH), 67.2 (CH–N), 61.1 (CH–Cl), 37.3 (CH_2_).

###### 4-(4-Bromophenyl)-3-chloro-1-(4-(2,7-dichloro-9*H*-fluoren-4-yl)thiazol-2-yl)azetidin-2-one (**6d**)

Pale yellow crystals, yield (87%), m.p. 112–114 °C; FT-IR (KBr): ν (cm^−1^) 3099 (CH arom.), 2955 (CH aliph.), 1792 (C=O), 1665 (C=N); ^1^H NMR (DMSO-*d*_6_): δ 7.83 (s, 1H, Flu-H), 7.76–7.65 (m, 4H, Flu-H & Ph-H), 7.50 (s, 1H, Flu-H), 7.40 (s, 1H, Flu-H), 7.27–7.22 (m, 3H, Flu-H & Thiazole-H), 7.12 (d, *J* = 8.0 Hz, 1H, CH–N), 4.27 (d, *J* = 8.0 Hz, 1H, CH–Cl), 4.02 (s, 2H, CH_2_); ^13^C NMR (100 MHz, DMSO-*d*_6_): δ 170.9 (C=O), 165.7 (C=N), 158.0 (C), 147.8 (C), 146.7 (C), 146.1 (C), 138.8 (C), 136.6 (C), 135.0 (C), 132.7 (CH), 132.6 (CH), 131.5 (C), 129.3 (C), 129.0 (CH), 127.4 (C), 127.1 (CH), 124.6 (CH), 124.5 (CH), 113.3 (CH), 107.4 (Thiazole-CH), 66.1 (CH–N), 54.1 (CH–Cl), 37.2 (CH_2_).

###### 3-Chloro-1-(4-(2,7-dichloro-9*H*-fluoren-4-yl)thiazol-2-yl)-4-(4-(dimethylamino)phenyl)-azetidin-2-one (**6e**)

Red crystals, yield (65%), m.p. 110–111 °C; FT-IR (KBr): ν (cm^−1^) 3069 (CH arom.), 2949 (CH aliph.), 1695(C=O), 1551 (C=N); ^1^H NMR (400 MHz, DMSO-*d*_6_) δ 7.93 (d, *J* = 8.0 Hz, 1H, Flu-H), 7.77–7.60 (m, 4H, Flu-H & Ph-H), 7.51 (s, 1H, Flu-H), 7.40 (s, 1H, Flu-H), 7.28 (d, *J* = 8.0 Hz, 2H, Ph-H), 7.22 (s, 1H, Thiazole-H), 6.80 (d, *J* = 4.0 Hz, 1H, CH–N), 4.19 (d, *J* = 4.0 Hz, 1H, CH), 4.02 (s, 2H, CH_2_), 3.02 (s, 6H, 2CH_3_); ^13^C NMR (100 MHz, DMSO-*d*_6_): δ 169.1 (C=O), 165.7 (C=O), 158.0 (C), 147.9 (C), 146.8 (C), 146.3 (C), 138.8 (C), 137.0 (C), 134.1 (C), 132.5 (C), 131.7 (C), 129.0 (CH), 127.1 (CH), 125.5 (CH), 124.7 (CH), 122.9 (CH), 122.2 (CH), 119.9 (CH), 113.31 (CH), 112.3 (CH), 112.0 (Thiazole-CH), 66.3 (CH–N), 62.1 (CH–Cl), 41.9 (CH_3_), 36.7 (CH_2_).

###### 3-Chloro-1-(4-(2,7-dichloro-9*H*-fluoren-4-yl)thiazol-2-yl)-4-(4-nitrophenyl)azetidin-2-one (**6f**)

Pale brown crystals, yield (89%), m.p. 114–116 °C; FT-IR (KBr): ν (cm^−1^) 3071 (CH arom.), 2954 (CH aliph.), 1691 (C=O), 1591 (C=N); ^1^H NMR (400 MHz, DMSO-*d*_6_): δ 8.17 (d, *J* = 8.0 Hz, 2H, Ph-H), 7.72 (d, *J* = 8.0 Hz, 1H, Flu-H), 7.66 (s, 1H, Flu-H), 7.51 (s, 1H, Flu-H), 7.44–7.40 (m, 3H, Ph-H & Flu-H), 7.27 (s, 1H, Flu-H), 7.21 (s, 1H, Thiazole-H), 7.02 (d, *J* = 8.0 Hz, 1H, CH–N), 4.28 (d, *J* = 8.0 Hz, 1H, CH–Cl), 4.03 (s, 2H, CH_2_); ^13^C NMR (100 MHz, DMSO-*d*_6_): δ 169.1 (C=O), 165.8 (C=N), 158.0 (C), 147.8 (C), 146.8 (C), 146.3 (C), 140.5 (C), 138.8 (C), 137.0 (C), 132.5 (C), 132.1 (C), 129.3 (C), 129.0 (CH), 127.5 (C), 127.10 (CH), 125.8 (CH), 125.5 (CH), 124.7 (CH), 124.5 (CH), 124.1 (CH), 113.3 (Thiazole-CH), 66.8 (CH–N), 61.6 (CH–Cl), 36.9 (CH_2_).

###### 4-(3-Chloro-1-(4-(2,7-dichloro-9*H*-fluoren-4-yl)thiazol-2-yl)-4-oxoazetidin-2-yl)benzoic acid (**6g**)

Pale yellow crystals, yield (76%), m.p. 125–127 °C; FT-IR (KBr): ν (cm^−1^) 3366 (OH), 3099 (CH arom.), 2956 (CH aliph.), 1691 (C=O), 1546 (C=N); ^1^H NMR (400 MHz, DMSO-*d*_6_) δ 12.73 (s, 1H, OH), 8.03 (d, *J* = 8.0 Hz, 2H, Ph-H), 7.75 (d, *J* = 8.0 Hz, 1H, Flu-H), 7.70 (s, 1H, Flu-H), 7.64 (s, 1H, Flu-H), 7.52 (m, 2H, Flu-H), 7.43–7.39 (m, 2H, Ph-H), 7.26 (s, 1H, Thiazole-H), 7.21 (d, *J* = 12.0 Hz, 1H, CH–N), 4.26 (d, J = 12.0 Hz, 1H, CH–Cl), 4.01 (s, 2H, CH_2_); ^13^C NMR (100 MHz, DMSO-*d*_6_): δ 169.1 (C=O), 165.8 (C=O), 158.7 (C=N), 148.2 (C), 146.8 (C), 146.2 (C), 138.8 (C), 137.0 (C), 132.5 (C), 132.1 (C), 131.7 (C), 130.4 (C), 130.0 (C), 128.9 (CH), 127.5 (CH), 127.08 (CH), 125.5 (CH), 124.6 (CH), 122.9 (CH), 122.1 (CH), 113.3 (Thiazole-CH), 65.9 (CH–N), 60.1 (CH–Cl), 36.9 (CH_2_).

###### 3-Chloro-1-(4-(2,7-dichloro-9*H*-fluoren-4-yl)thiazol-2-yl)-4-(4-hydroxy-3-methoxyphenyl)-azetidin-2-one (**8h**)

Pale yellow crystals, yield (84%), m.p. 95–97 °C; FT-IR (KBr): ν (cm^−1^) 3365 (OH), 3067 (CH arom.), 2954 (CH aliph.), 1691 (C=O), 1546 (C=N); ^1^H NMR (400 MHz, DMSO-*d*_6_): δ 7.90 (d, J = 8.0 Hz, 1H, Flu-H), 7.77–7.65 (m, 3H, Flu-H & Ph-H), 7.51 (s, 1H, Flu-H), 7.45 (d, *J* = 8.0 Hz, 1H, Flu-H), 7.40 (s, 1H, Ph-H), 7.27 (m, 2H, Ph-H & Thiazole-H), 7.21 (d, J = 12.0 Hz, 1H, CH–N), 6.97 (s, 1H, OH), 4.45 (s, 3H, CH_3_), 4.28 (d, J = 12.0 Hz, 1H, CH–Cl), 4.02 (s, 2H, CH_2_); ^13^C NMR (100 MHz, DMSO-*d*_6_): δ 169.1 (C=O), 165.8 (C=N), 158.0 (C), 157.1 (C), 147.9 (C), 146.8 (C), 146.3 (C), 138.8 (C), 137.0 (C), 132.5 (C), 132.1 (C), 131.7 (C), 129.0 (CH), 127.7 (CH), 127.1 (CH), 125.8 (CH), 125.5 (CH), 124.6 (CH), 122.9 (CH), 122.1 (CH), 113.3 (Thiazole-CH), 67.2 (CH–N), 61.0 (CH–Cl), 56.8 (CH_3_), 36.9 (CH_2_).

###### 3-Chloro-1-(4-(2,7-dichloro-9*H*-fluoren-4-yl)thiazol-2-yl)-4-styrylazetidin-2-one (**6i**)

Pale brown crystals, yield (60%), m.p. 110–112 °C; FT-IR (KBr): ν (cm^−1^) 3062 (CH arom.), 2951 (CH aliph.), 1702 (C=O), 1542 (C=N); ^1^H NMR (400 MHz, DMS-*d*_6_) δ 7.80 (s, 1H, Flu-H), 7.65–7.60 (m, 2H, Flu-H), 7.52 (d, *J* = 4.0 Hz, 1H, Flu-H), 7.34 (s, 1H, Flu-H), 7.30–7.27 (m, 3H, Ph-H), 7.21–7.16 (m, 3H, Ph-H & Thiazole-H), 7.12 (d, *J* = 12.0 Hz, 1H, CH–N), 6.80–6.73 (m, 2H, CH=CH), 4.40 (d, *J *= 12.0 Hz, 1H, CH–Cl), 4.01 (s, 2H, CH_2_); ^13^C NMR (100 MHz, DMSO-*d*_6_): δ 169.1 (C=O), 165.8 (C=N), 158.0 (C), 147.8 (C), 146.8 (C), 146.3 (C), 138.8 (C), 137.0 (C), 132.5 (C), 132.1 (C), 131.6 (C), 129.57 (CH), 129.0 (CH), 128.4 (CH), 127.7 (CH), 127.1 (CH), 125.5 (CH), 124.6 (CH), 123.1 (CH), 122.8 (CH), 121.4 (CH), 113.3 (Thiazole-CH), 68.0 (CH–N), 61.3 (CH–Cl), 36.4 (CH_2_).

###### 3-Chloro-1-(4-(2,7-dichloro-9*H*-fluoren-4-yl)thiazol-2-yl)-4-(furan-2-yl)azetidin-2-one (**6j**)

Pale yellow crystals, yield (95%), m.p. 95–98 °C; FT-IR (KBr): ν (cm^−1^) 3056 (CH arom.), 2952 (CH aliph.), 1695 (C=O), 1544 (C=N); ^1^H NMR (400 MHz, DMSO-*d*_6_): δ 7.90 (d, *J* = 8.0 Hz, 1H, Flu-H), 7.77 (s, 1H, Flu-H), 7.66 (s, 1H, Flu-H), 7.51 (d, *J* = 4.0 Hz, 1H, Furan-H), 7.46–7.40 (m, 3H, Flu-H & Furan-H), 7.28 (d, *J* = 8.0 Hz, 1H, Flu-H), 7.20 (s, 1H, Thiazole-H), 7.11 (d, *J* = 12.0 Hz, 1H, CH–N), 4.22 (d, *J* = 12.0 Hz, 1H, CH–Cl), 4.02 (s, 2H, CH_2_); ^13^C NMR (100 MHz, DMSO-*d*_6_): δ 168.4 (C=O), 165.7 (C=N), 158.9 (C), 148.4 (C), 146.8 (C), 146.3 (C), 138.8 (C), 137.0 (C), 132.5 (C), 132.1 (C), 131.7 (C), 129.0 (CH), 127.7 (CH), 127.10 (CH), 125.9 (CH), 125.5 (CH), 124.6 (CH), 122.9 (CH), 122.1 (CH), 113.3 (Thiazole-CH), 66.7 (CH–N), 62.0 (CH–Cl), 37.3 (CH_2_).

###### 3-Chloro-1-(4-(2,7-dichloro-9*H*-fluoren-4-yl)thiazol-2-yl)-4-(thiophen-2-yl)azetidin-2-one (**6k**)

Yellow crystals, yield (51%), m.p. 109–111 °C; FT-IR (KBr): ν (cm^−1^) 3109 (CH arom.), 2951 (CH aliph.), 1691 (C=O), 1646 (C=N); ^1^H NMR (400 MHz, DMSO-*d*_6_) δ 7.92 (s, 1H, Flu-H), 7.72–7.55 (m, 2H, Flu-H & Thienyl-H), 7.51 (s, 1H, Flu-H), 7.44 (s, 1H, Flu-H), 7.40–7.32 (m, 2H, Thienyl-H & Thiazole-H), 7.28 (d, *J* = 4.0 Hz, 1H, Flu-H), 7.21 (d, *J* = 4.0 Hz, 1H, Thienyl-H), 7.06 (d, *J* = 12.0 Hz, 1H, CH–N), 4.28 (d, *J* = 12.0 Hz, 1H, CH–Cl), 4.03 (s, 2H, CH_2_); ^13^C NMR (100 MHz, DMSO-*d*_6_): δ 169.1 (C=O), 165.8 (C=N), 158.9 (C), 147.8 (C), 146.8 (C), 146.3 (C), 142.1 (C), 138.8 (C), 137.0 (C), 132.3 (C), 131.7 (C), 129.0 (C), 128.9 (CH), 128.3 (CH), 127.1 (CH), 125.6 (CH), 124.6 (CH), 123.4 (CH), 121.2 (CH), 113.6 (CH), 112.4 (Thiazole-CH), 71.1 (CH–N), 61.0 (CH–Cl), 37.3 (CH_2_).

###### 3-Chloro-1-(4-(2,7-dichloro-9*H*-fluoren-4-yl)thiazol-2-yl)-4-(1*H*-pyrrol-2-yl)azetidin-2-one (**6l**)

Green crystals, yield (93%), m.p. 105–107 °C; FT-IR (KBr): ν (cm^−1^) 3046 (CH arom.), 2954 (CH aliph.), 1705 (C=O), 1695 (C=N); ^1^H NMR (400 MHz, DMSO-*d*_6_): δ 7.78 (s, 1H, Flu-H), 7.73–7.66 (m, 2H, Flu-H), 7.55–7.51 (m, 2H, Flu-H & Pyrrole-H), 7.46 (d, *J* = 8.0 Hz, 1H, Flu-H), 7.41 (s, 1H, Thiazole-H), 7.29–7.27 (m, 2H, Pyrrole-H), 7.21 (d, *J* = 8.0 Hz, 1H, CH–N), 4.45 (s, 1H, NH), 4.28 (d, *J* = 8.0 Hz, 1H, CH–Cl), 4.03 (s, 2H, CH_2_); ^13^C NMR (100 MHz, DMSO-*d*_6_): δ 169.1 (C=O), 166.6 (C=N), 158.0 (C), 157.1 (C), 147.8 (C), 146.8 (C), 146.3 (C), 138.8 (C), 137.0 (C), 132.5 (C), 131.7 (C), 129.0 (CH), 127.7 (CH), 127.11 (CH), 125.9 (CH), 125.6 (CH), 124.6 (CH), 122.9 (CH), 122.2 (CH), 113.3 (Thiazole-CH), 67.0 (CH–N), 61.9 (CH–Cl), 36.9 (CH_2_).

###### 3-Chloro-1-(4-(2,7-dichloro-9*H*-fluoren-4-yl)thiazol-2-yl)-4-(pyridin-4-yl)azetidin-2-one (**6m**)

Pale yellow crystals, yield (56%), m.p. 249–250 °C; FT-IR (KBr): ν (cm^−1^) 3096 (CH arom.), 2927 (CH aliph.),1772 (C=O), 1686 (C=N), 1597 (C=N); ^1^H NMR (400 MHz, DMSO-*d*_6_): δ 7.89 (s, 1H, Flu-H), 7.65 (d, *J* = 4.0 Hz, 2H, Pyridine-H), 7.44 (d, *J* = 8.0 Hz, 1H, Flu-H), 7.40 (m, 1H, Flu-H), 7.32 (s, 1H, Flu-H), 7.27–7.20 (m, 3H, Flu-H & Pyridine-H), 7.11 (s, 1H, Thiazole-H), 7.02 (d, *J* = 12.0 Hz, 1H, CH–N), 4.27 (d, *J* = 12.0 Hz, 1H, CH–Cl), 4.01 (s, 2H, CH_2_); ^13^C NMR (100 MHz, DMSO-*d*_6_): δ 169.1 (C=O), 167.9 (C=N), 165.8 (C=N), 158.0 (C), 147.8 (CH), 146.5 (C), 146.3 (C), 138.8 (C), 137.0 (C), 132.2 (C), 132.0 (C), 131.6 (C), 128.9 (CH), 127.1 (CH), 125.6 (CH), 125.4 (CH), 124.6 (CH), 118.8 (CH), 116.8 (CH), 113.3 (Thiazole-CH), 70.1 (CH–N), 65.8 (CH–Cl), 36.9 (CH_2_).

###### 3-Chloro-1-(4-(2,7-dichloro-9*H*-fluoren-4-yl)thiazol-2-yl)-4-(quinolin-4-yl)azetidin-2-one (**5n**)

Orange crystals, yield (58%), m.p. 175–177 °C; FT-IR (KBr): ν (cm^−1^) 3068 (CH arom.), 2852 (CH aliph.), 1771 (C=O), 1683 (C=N), 1584 (C=N); ^1^H NMR (400 MHz, DMSO-*d*_6_): δ 9.39–9.30 (m, 1H, Quinoline-H), 8.59 (d, *J* = 8.0 Hz, 1H, Quinoline-H), 8.45 (d, *J* = 8.0 Hz, 1H, Quinolin-H), 8.38 (d, *J* = 8.0 Hz, 1H, Quinolin-H), 8.27 (d, *J* = 12.0 Hz, 1H, Quinolin-H), 8.21–8.19 (m, 1H, Quinolin-H), 7.93 (s, 1H, Flu-H), 7.66–7.53 (m, 2H, Flu-H), 7.41 (s, 1H, Flu-H), 7.29–7.14 (m, 2H, Flu-H & Thiazole-H), 7.06 (m, 1H, CH–N), 4.27 (m, 1H, CH–Cl), 4.02 (s, 2H,CH_2_); ^13^C NMR (100 MHz, DMSO-*d*_6_): δ 169.1 (C=O), 167.9 (C=N), 165.8 (C=N), 158.2 (CH), 158.0 (C), 147.1 (C), 146.9 (C), 146.3 (C), 141.6 (C), 140.5 (C), 138.7 (C), 134.7 (C), 133.0 (C), 128.7 (CH), 127.1 (CH), 126.8 (CH), 125.5 (CH), 125.1 (CH), 124.6 (CH), 124.5 (CH), 123.7 (CH), 123.5 (CH), 121.8 (CH), 113.9 (Thiazole-CH), 67.0 (CH–N), 62.5 (CH–Cl), 34.5 (CH_2_).

### Antimicrobial screening

#### Used microorganisms

All microbial strains were kindly provided from the department of Medical Microbiology and Immunology faculty of Medicine Assiut University, these clinical isolates were obtained from clinical cases of infections admitted to Assiut University hospital as urinary tract infections, corneal ulcers, bacterial and fungal pneumonia, otomycosis, oral thrush and wound infections. The clinical isolates were proved by using the VITEK 2 automated microbiology system (BioMérieux).

The clinical isolates used were multidrug resistant strains, they were resistant to β lactam (penicillin, amoxacillin, oxacillin), cephalosporins (cefazolin, cefaclor and cefepime) and macrolides (erythromycin and clarithromycin), they included Gram positive bacteria as *Staphylococcus aureus* (*S. aureus*), Methicillin-resistant *Staphylococcus aureus* (MRSA), *Streptococcus pneumoniae* (*S. pneumoniae*), and Gram negative bacteria as *Escherichia coli* (*E. coli*), *Klebsiella pneumoniae* (*K. pneumoniae*), *Pseudomonas aeruginosa* (*P. aeruginosa*), and *Acinetobacter baumannii* (*A. baumannii*). The fungal strains that were tested are *Aspergillus flavus* (*A. flavus*), *A. niger* (*A. niger*) *and Candida albicans* (*C. albicans*).

#### Initial evaluation of the fluorene derivatives antibacterial and antifungal activity

The antimicrobial activity of the fluorene derivatives was initially evaluated by agar well diffusion assay [40]. Mueller–Hinton agar (CM0337) was poured into Petri dishes at 50–60 °C and left to solidify for 15 min. Subsequently, overnight microbial suspensions of tested strains was adjusted to turbidity of 0.5 McFarland Standard, which equals to 1–2 × 10^8^ CFU/mL for bacteria and 1–5 × 10^6^ for fungi. The microbial inoculums were then diluted in 1:100 ratio in case of bacteria and 1:10 ratio in case of fungi in order to get 1–5 × 10^5^ CFU/mL. a sterile cotton swab was dipped into the adjusted microbial suspension and the Mueller–Hinton agar plates were inoculated by evenly streaking cotton swab over the agar medium. Then wells with a diameter of 0.5 cm were cut in the medium with a sterile cork borer. Stock solutions of the flourene derivatives were diluted in DMSO 1% to get 500 μg/mL concentrations. The tested flourene derivatives and controls (50 μL) were dispensed into the wells. The plates were incubated for 24 h at 37 °C for bacteria and *C. albicans* while at 25 °C for *A. falvus* and *A. niger*. The diameters of zones of inhibition (ZOI) around the wells were measured in mm. Following control agents were used: positive control agents—vancomycin (50 μg/mL) for Gram positive bacteria, gentamicin (10 μg/mL) (for Gram negative bacteria) and fluconazole 25 μg/mL for fungi and negative control agent is 1% DMSO.

#### Determination of MIC values for the most active fluorene derivatives

Determination of Minimum inhibitory concentrations (MIC) of flourene derivatives was done using broth microdilution method [41]. The procedure involved preparation of twofold dilutions of the fluorene derivatives ranging from (500–7.8 μg/mL) in sterile Mueller–Hinton broth inside the wells of 96-well microplate (Sarstedt, Germany). The inoculums of test strains prepared from fresh overnight cultures were adjusted to 0.5 McFarland standards, which equals to 1–2 × 10^8^ CFU/mL for bacteria, the procedure was done according to CLSI 2012 [42]. The highest dilution of samples (flourene derivatives) without visible growth after 24 h incubation at 37 °C was considered as MIC. For this assay the positive control agents were vancomycin (range: 0.7–50 μg/mL), gentamicin (range: 0.15–10 μg/mL) and the negative control was 1% DMSO.

For proper determination of the MIC end point resazurin dye has been used. A stock solution of resazurin sodium salt powder (Titan Biotech) was prepared at 0.02% (wt/vol) in distilled water, sterilized by filtration through a 0–2 µm filter into a sterile light protected container then stored protected from light at 4 °C for up to 1 week, or at − 20% for long term use, then 10–15% resazurin solution of the total volume in wells was added to each well and incubation for 1–4 h at 37 °C was done. A change in color from blue to pink indicates the growth of bacteria, and MIC was defined as the lowest concentration of the drug that prevented this change in color.

##### Data processing

All experiments were independently repeated three times. Obtained data were processed; standard deviations were calculated using GraphPad Prism 5.03 (GraphPad Software, Inc.; USA) software.

Media and reagents:

- Muller Hinton agar oxoid code: CM0337
- Muller Hinton broth oxoid code: CM 0405
- Mannitol salt agar oxoid code: CM 0151
- Columbia agar oxoid code: CM 0331
- Orsab oxoid code CM 1008
- Nutrient agar oxoid code: CM0003
- Eosin methylene Blue Himedia M317

Equipment:

- Petri dishes
- Crock borer
- Sterile syringe needle and swabs
- Microtitre plates
- Micropipette
- Sterile tips

### Cytotoxicity screening

#### Cell culture

WI-38 normal lung fibroblast cells, A549 lung cancer cells, and MDA-MB-231 breast cancer cells were obtained from VACSERA—Cell Culture Unit, Cairo, Egypt. The cell lines were originally obtained from the American Tissue Culture Collection (ATCC). WI-38, A549, and MDA-MB-231 cell lines were cultured in RPMI-1640 medium supplemented with 10% inactivate fetal bovine serum (FBS) and 1% penicillin/streptomycin were bought (Gibco, Invitrogen, CA).

#### Cell viability assay

WI-38, A549, and MDA-MB-231 cells were seeded into 96-well plates (at a density of 5000 cells/well). On the following day, cells were treated with different concentrations (0, 1, 10, 31.25, 62.5, 125, 250, 500 µg/mL) of 16 fluorene derivatives in fresh medium and incubated for another 24 h. Cell viability was then assessed using the MTT assay (Sigma Aldrich, St. Louis, MO, USA), and the absorbance was read at 570 nM using an ELISA microplate reader (Molecular Devices, Downingtown, PA, USA).

#### FACS analysis

To uncover the mechanism of cell death for the compounds **5h**, **6c**, **6d** and **6k** on WI-38, A549, and MDA-MB-231 cells; Annexin v and propedium Iodide (PI) were used. In brief, WI-38, A549, MDA-MB-231 cells were cultured in 10 tissue culture dish with initial number 4 × 10^5^ cell/Ml in RPMI growth media. In the following day, cells were treated with **6c**, **6d** and **6k** as the following; (0.0, 500 µg/mL form each drug for WI-38 treatment, 0.0, 85, 117 and 200 µg/mL; respectively, for A5489 and 0.0, 250 from each for MDA-MB-231 cells treatment). After 24 h incubations, cells were washed and trypsinized and suspended in 50 µL 1X Annexin v binding buffer followed by adding 5 µL FITC Annexin V and incubated for 15 min at room temperature then 5 µL of PI were added to each tube. Finally, 400 µL of 1X Annexin v binding buffer were added to each tube and analyzed using Becton–Dickinson FACS Caliber.

## Supplementary information

**Additional file 1:** NMR spectra, docking and molecular modeling calculations of invetigated bioactive fluorenes.

## Data Availability

Additional information with NMR spectra and molecular docking are attached.

## Abbreviations

FACS: Fluorescence-activated cell sorting
HIV: Human immunodeficiency virus
DHFR: Dihydrofolate reductase
NADPH: Nicotinamide adenine dinucleotide phosphate
DHF: 7,8-Dihydrofolate
THF: 5,6,7,8-Tetrahydrofolate
DNA: Deoxyribonucleic acid
DCM: Dichloromethane
THF: Tetrahydrofuran
DCC: *N*,*N*′-Dicyclohexylcarbodiimide
DMF: Dimethylformamide
FT-IR: Fourier transform infrared
1H NMR: Proton nuclear magnetic resonance
13C NMR: Carbon-13 nuclear magnetic resonance
DEPT-135: Distortionless enhancement by polarization transfer
ZOI: Zone of inhibition
*S. aureus*: *Staphylococcus aureus*
MRSA: Methicillin-resistant *Staphylococcus aureus*
*S. pneumoniae*: *Streptococcus pneumoniae*
*E. coli*: *Escherichia coli*
*K. pneumoniae*: *Klebsiella pneumoniae*
*P. aeruginosa*: *Pseudomonas aeruginosa*
*A. baumannii*: *Acinetobacter baumannii*
*A. flavus*: *Aspergillus flavus*
*A. niger*: *Aspergillus niger*
*C. albicans*: *Candida albicans*
MIC: The minimum inhibitory concentration
DMSO: Dimethyl sulphoxide
MOE: Molecular Operating Environment
CCG: Chemical Computing Group
RMSD: The root-mean-square deviation
