# Supplementary material for: Bioactive fluorenes. Part III: 2,7-dichloro-9H-fluorene-based thiazolidinone and azetidinone analogues as anticancer and antimicrobial against multidrug resistant strains agents
Source: BMC Chem. 2020 Jun 25;14(1):42. doi: 10.1186/s13065-020-00694-2 (PMC7315563; doi:10.1186/s13065-020-00694-2)
Supplement: Supplementary file 1 — Additional file 1: NMR spectra, docking and molecular modeling calculations of invetigated bioactive fluorenes. [file 13065_2020_694_MOESM1_ESM.docx]

# ADDITIONAL INFORMATION FOR

**Bioactive Fluorenes. Part III. 2,7-Dichloro-9*H*-fluorene-based thiazolidinone and azetidinone analogues as anticancer and antimicrobial against multidrug resistant strains agents**

Essam M. Hussein^1,2^, [Reem I. Alsantali](javascript:__doPostBack('ctl00$Body$ucUniProposal$gvParticipants$ctl02$lbUserNameEn',''))^1,3^, Moataz Morad^1^, Rami J. Obaid^1^, Hatem M. Altass^1^, Ali Sayqal^1^, Mohamed A. S. Abourehab^4,5^, Amal A. Elkhawaga^6^, Ahmed S. M. Aboraia^7^, Saleh A. Ahmed^1,2*^

*^1^ Department of Chemistry, Faculty of Applied Science, Umm Al-Qura University, 21955 Saudi Arabia*

*^2^ Chemistry Department, Faculty of Science, Assiut University, 71516 Assiut, Egypt*

*^3^ Department of Pharmaceutical Chemistry, Pharmacy College, Taif University, 888-Taif, Saudi Arabia*

*^4^ Department of Pharmaceutics, Faculty of Pharmacy, Umm Al-Qura University, Makkah, Saudi Arabia*

*^5^ Department of Pharmaceutics and Industrial Pharmacy, Faculty of Pharmacy, Minia University, Minia, Egypt*

*^6^ Department of Medical Microbiology & Immunology, Faculty of Medicine Assiut University, 71516 Assiut, Egypt*

*^7^Department of Medicinal Chemistry, Faculty of Pharmacy, Assiut University, 71516 Assiut, Egypt*

*Correspondence may be addressed to:

Saleh A. Ahmed: E-mail address: [saahmed@uqu.edu.sa](mailto:saahmed@uqu.edu.sa), [saleh_63@hotmail.com](mailto:saleh_63@hotmail.com)

1. **Spectral data of the new synthesized compounds**

**^1^H NMR spectrum of *N*-benzylidene-4-(2,7-dichloro-9*H*-fluoren-4-yl)thiazol-2-amine (4a)**

**^13^C NMR spectrum of *N*-benzylidene-4-(2,7-dichloro-9*H*-fluoren-4-yl)thiazol-2-amine (4a)**

**^1^H NMR spectrum of *N*-(4-bromobenzylidene)-4-(2,7-dichloro-9*H*-fluoren-4-yl)thiazol-2-amine (4d)**

**^13^C NMR spectrum of *N*-(4-bromobenzylidene)-4-(2,7-dichloro-9*H*-fluoren-4-yl)thiazol-2-amine (4d)**

**^1^H NMR spectrum of 4-(2,7-dichloro-9H-fluoren-4-yl)-N-(4-(dimethylamino)benzylidene)thiazol-2-amine** (**4e**)

**^13^C NMR spectrum of 4-(2,7-dichloro-9H-fluoren-4-yl)-N-(4-(dimethylamino)benzylidene)thiazol-2-amine** (**4e**)

**^1^H NMR spectrum of 4-((4-(2,7-dichloro-9H-fluoren-4-yl)thiazol-2-ylimino)methyl)-2-methoxyphenol** (**4h**)

**^13^C NMR spectrum of 4-((4-(2,7-dichloro-9H-fluoren-4-yl)thiazol-2-ylimino)methyl)-2-methoxyphenol (4h)**

**^1^H NMR spectrum of 4-(2,7-dichloro-9H-fluoren-4-yl)-N-(furan-2-ylmethylene)thiazol-2-amine** (**4j**)

**^13^C NMR spectrum of 4-(2,7-dichloro-9H-fluoren-4-yl)-N-(furan-2-ylmethylene)thiazol-2-amine (4j)**

**^1^H NMR spectrum of 4-(2,7-dichloro-9H-fluoren-4-yl)-N-(thiophen-2-ylmethylene)thiazol-2-amine** (**4k**)

**^13^C NMR spectrum of 4-(2,7-dichloro-9H-fluoren-4-yl)-N-(thiophen-2-ylmethylene)thiazol-2-amine** (**4k**)

**^1^H NMR spectrum of N-((1H-pyrrol-2-yl)methylene)-4-(2,7-dichloro-9H-fluoren-4-yl)thiazol-2-amine** (**4l**)

**^13^C NMR spectrum of N-((1H-pyrrol-2-yl)methylene)-4-(2,7-dichloro-9H-fluoren-4-yl)thiazol-2-amine** (**4l**)

**^1^H NMR spectrum of 3-(4-(2,7-dichloro-9H-fluoren-4-yl)thiazol-2-yl)-2-phenylthiazolidin-4-one** (**5a**)

**^1^H NMR spectrum of 3-(4-(2,7-dichloro-9H-fluoren-4-yl)thiazol-2-yl)-2-(4-methoxyphenyl)thiazolidin-4-one** (**5b**)

**^1^H NMR spectrum of 2-(4-chlorophenyl)-3-(4-(2,7-dichloro-9H-fluoren-4-yl)thiazol-2-yl)thiazolidin-4-one (5c)**

**^1^H NMR spectrum of 2-(4-bromophenyl)-3-(4-(2,7-dichloro-9H-fluoren-4-yl)thiazol-2-yl)thiazolidin-4-one (5d)**

**^1^H NMR spectrum of 3-(4-(2,7-dichloro-9H-fluoren-4-yl)thiazol-2-yl)-2-(4-(dimethylamino)-phenyl)thiazolidin-4-one** (**5e**)

**^1^H NMR spectrum of 3-(4-(2,7-dichloro-9H-fluoren-4-yl)thiazol-2-yl)-2-(4-nitrophenyl)-thiazolidin-4-one** (**5f**)

**^1^H NMR spectrum of 4-(3-(4-(2,7-dichloro-9H-fluoren-4-yl)thiazol-2-yl)-4-oxothiazolidin-2-yl)benzoic acid** (**5g**)

**^1^H NMR spectrum of 3-(4-(2,7-dichloro-9H-fluoren-4-yl)thiazol-2-yl)-2-styrylthiazolidin-4-one (5i)**

**^1^H NMR spectrum of 3-(4-(2,7-dichloro-9H-fluoren-4-yl)thiazol-2-yl)-2-(furan-2-yl)thiazolidin-4-one** (**5j**)

**^1^H NMR spectrum of 3-(4-(2,7-dichloro-9H-fluoren-4-yl)thiazol-2-yl)-2-(thiophen-2-yl)thiazolidin-4-one (5k)**

**^13^C NMR spectrum of 3-(4-(2,7-dichloro-9H-fluoren-4-yl)thiazol-2-yl)-2-(thiophen-2-yl)thiazolidin-4-one (5k)**

**^1^H NMR spectrum of 3-(4-(2,7-dichloro-9H-fluoren-4-yl)thiazol-2-yl)-2-(1H-pyrrol-2-yl)thiazolidin-4-one (5l)**

**^1^H NMR spectrum of 3-(4-(2,7-dichloro-9H-fluoren-4-yl)thiazol-2-yl)-2-(pyridin-4-yl)thiazolidin-4-one (5m)**

**^1^H NMR spectrum of 3-(4-(2,7-dichloro-9H-fluoren-4-yl)thiazol-2-yl)-2-(pyridin-4-yl)thiazolidin-4-one (5m)**

**^1^H NMR spectrum of 3-(4-(2,7-dichloro-9H-fluoren-4-yl)thiazol-2-yl)-2-(quinolin-4-yl)thiazolidin-4-one (5n)**

**^13^C NMR spectrum of 3-(4-(2,7-dichloro-9H-fluoren-4-yl)thiazol-2-yl)-2-(quinolin-4-yl)thiazolidin-4-one (5n)**

**^1^H NMR spectrum of 3-chloro-1-(4-(2,7-dichloro-9H-fluoren-4-yl)thiazol-2-yl)-4-phenylazetidin-2-one (6a)**

**^1^H NMR spectrum of 3-chloro-1-(4-(2,7-dichloro-9H-fluoren-4-yl)thiazol-2-yl)-4-(4-methoxyphenyl)-azetidin-2-one (6b)**

**^13^C NMR spectrum of 3-chloro-1-(4-(2,7-dichloro-9H-fluoren-4-yl)thiazol-2-yl)-4-(4-methoxyphenyl)-azetidin-2-one (6b)**

**
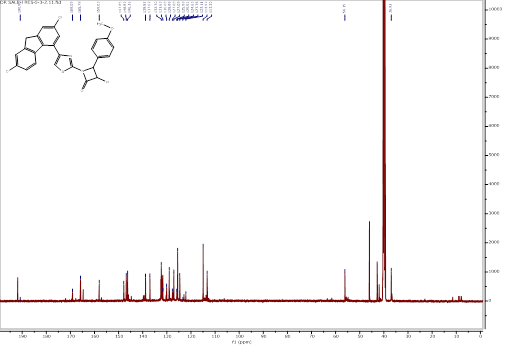
**

**^1^H NMR spectrum of 3-chloro-4-(4-chlorophenyl)-1-(4-(2,7-dichloro-9H-fluoren-4-yl)thiazol-2-yl)azetidin-2-one** (**6c**)

**^13^C NMR spectrum of 3-chloro-4-(4-chlorophenyl)-1-(4-(2,7-dichloro-9H-fluoren-4-yl)thiazol-2-yl)azetidin-2-one** (**6c**)

**^1^H NMR spectrum of 4-(4-bromophenyl)-3-chloro-1-(4-(2,7-dichloro-9H-fluoren-4-yl)thiazol-2-yl)azetidin-2-one** (**6d**)

**^13^C NMR spectrum of 4-(4-bromophenyl)-3-chloro-1-(4-(2,7-dichloro-9H-fluoren-4-yl)thiazol-2-yl)azetidin-2-one** (**6d**)

**^1^H NMR spectrum of 3-chloro-1-(4-(2,7-dichloro-9H-fluoren-4-yl)thiazol-2-yl)-4-(4-(dimethylamino)-phenyl)azetidin-2-one (6e)**

**^13^C NMR spectrum of 3-chloro-1-(4-(2,7-dichloro-9H-fluoren-4-yl)thiazol-2-yl)-4-(4-(dimethylamino)-phenyl)azetidin-2-one (6e)**

**^1^H NMR spectrum of 3-chloro-1-(4-(2,7-dichloro-9H-fluoren-4-yl)thiazol-2-yl)-4-(4-nitrophenyl)-azetidin-2-one** (**6f**)

**^1^H NMR spectrum of 4-(3-chloro-1-(4-(2,7-dichloro-9H-fluoren-4-yl)thiazol-2-yl)-4-oxoazetidin-2-yl)benzoic acid (6g)**

**^13^C NMR spectrum of 4-(3-chloro-1-(4-(2,7-dichloro-9H-fluoren-4-yl)thiazol-2-yl)-4-oxoazetidin-2-yl)benzoic acid (6g)**

**^1^H NMR spectrum of 3-chloro-1-(4-(2,7-dichloro-9H-fluoren-4-yl)thiazol-2-yl)-4-(4-hydroxy-3-methoxyphenyl)azetidin-2-one (6h)**

**^1^H NMR spectrum of 3-chloro-1-(4-(2,7-dichloro-9H-fluoren-4-yl)thiazol-2-yl)-4-(furan-2-yl)azetidin-2-one (6j)**

**^13^C NMR spectrum of 3-chloro-1-(4-(2,7-dichloro-9H-fluoren-4-yl)thiazol-2-yl)-4-(quinolin-4-yl)azetidin-2-one (6n)**

1. **Docking and Molecular Modeling Calculations**

**2D and 3D representation of the synthesized compounds interaction with DNA topoisomerase I enzyme**

| 5b  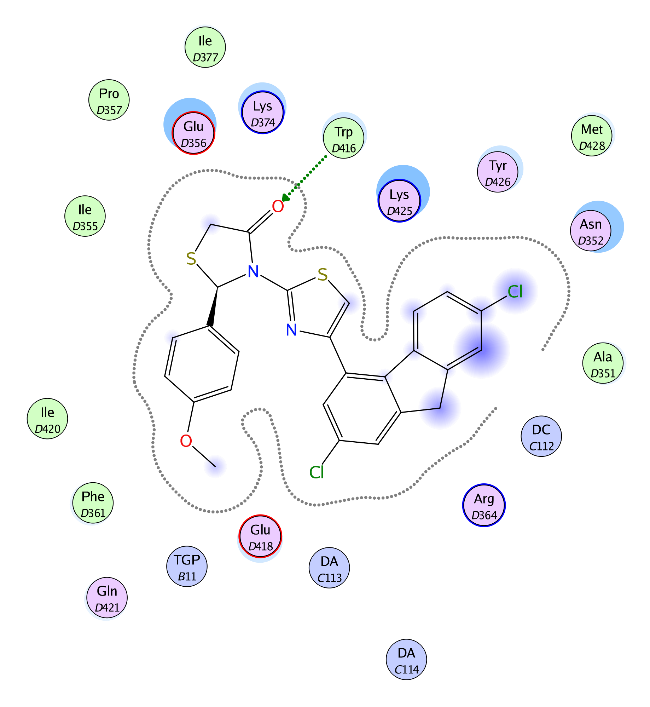 | 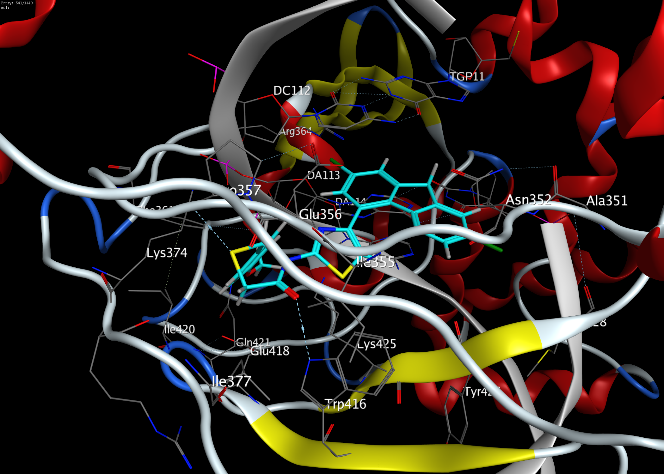 |
| --- | --- |
| 5c  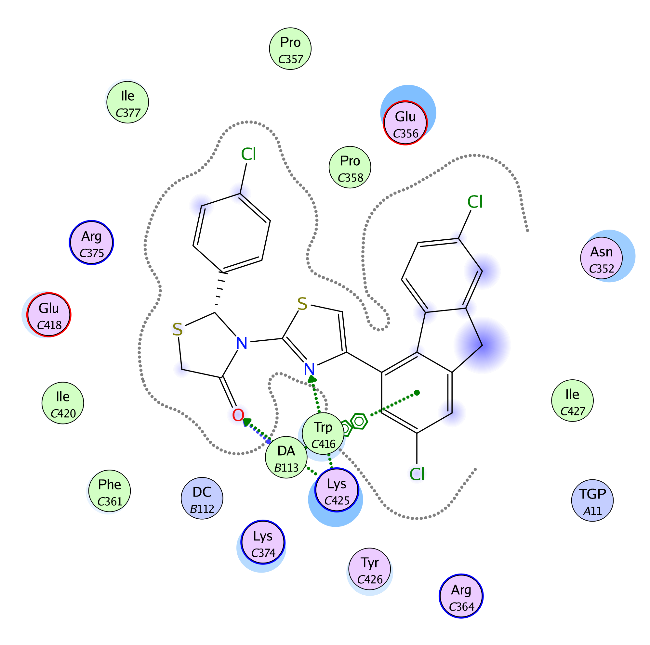 | **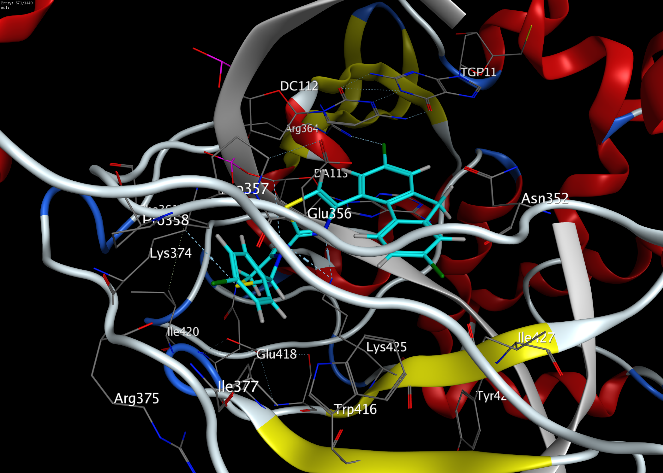** |
| 5d  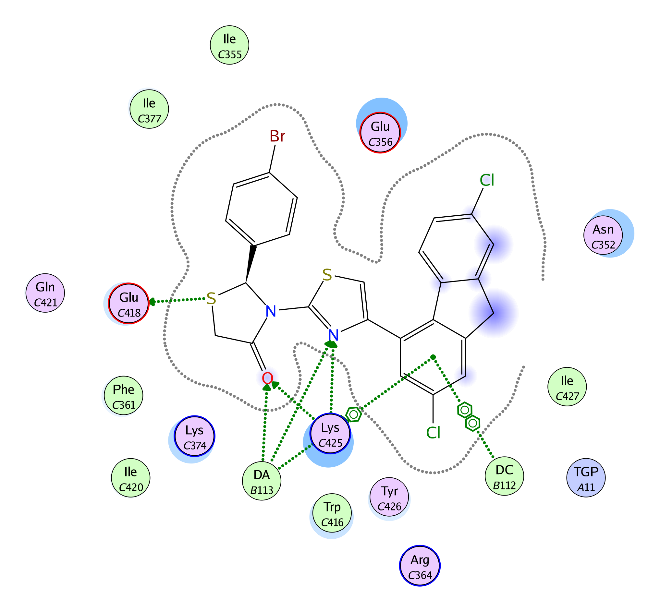 | **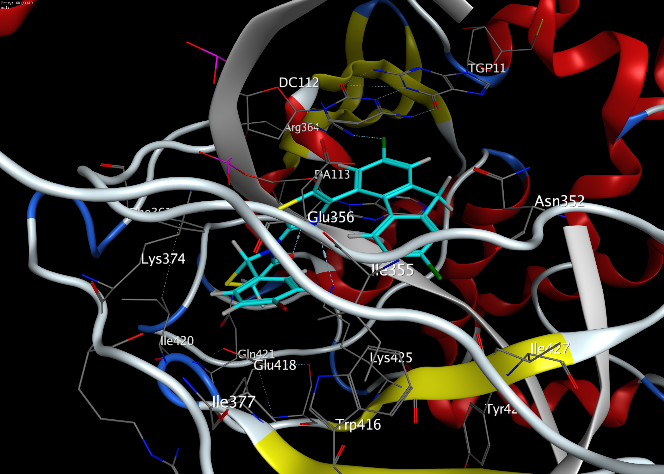** |
| 5e  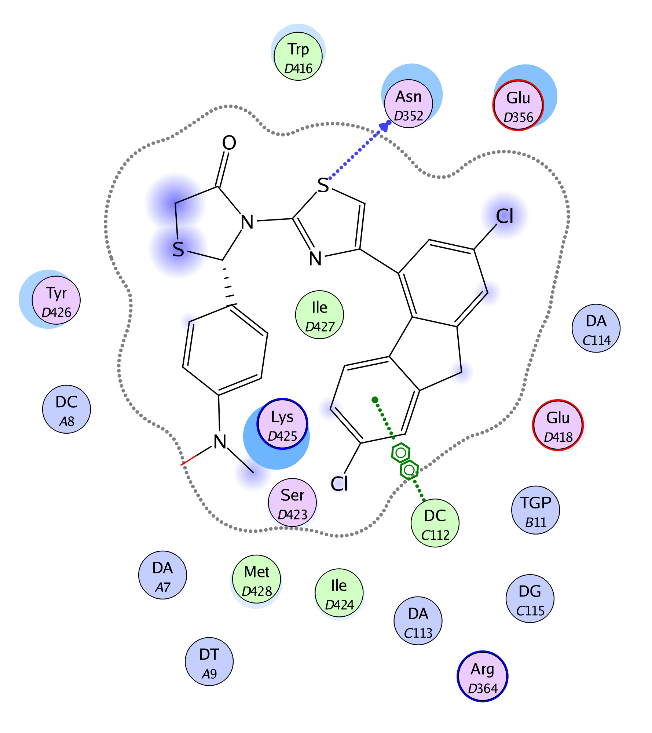 | **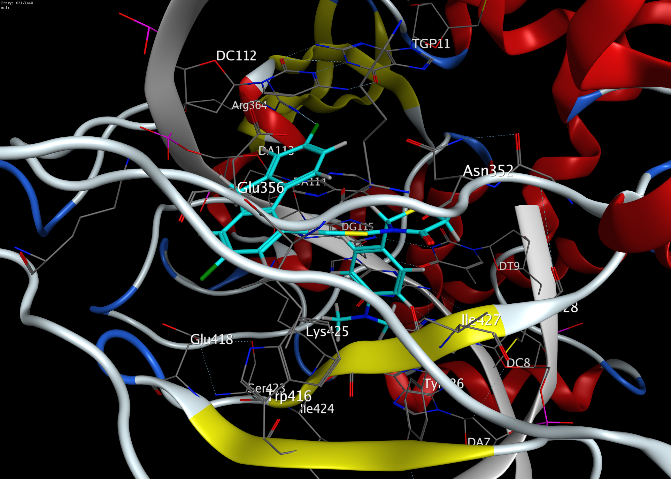** |
| 5f  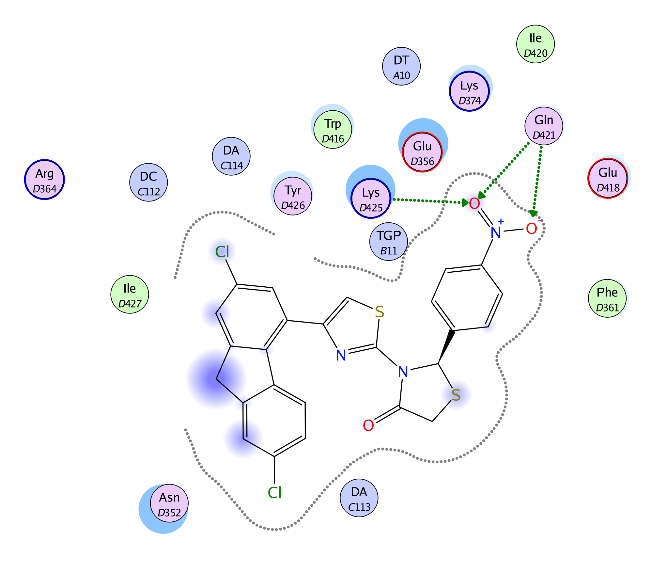 | **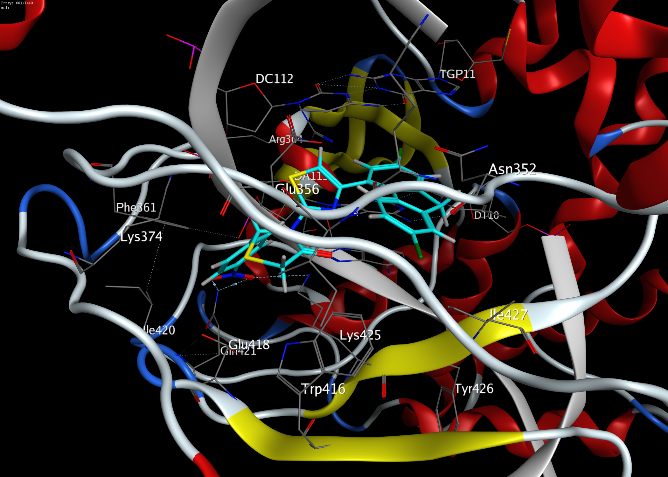** |
| 5g  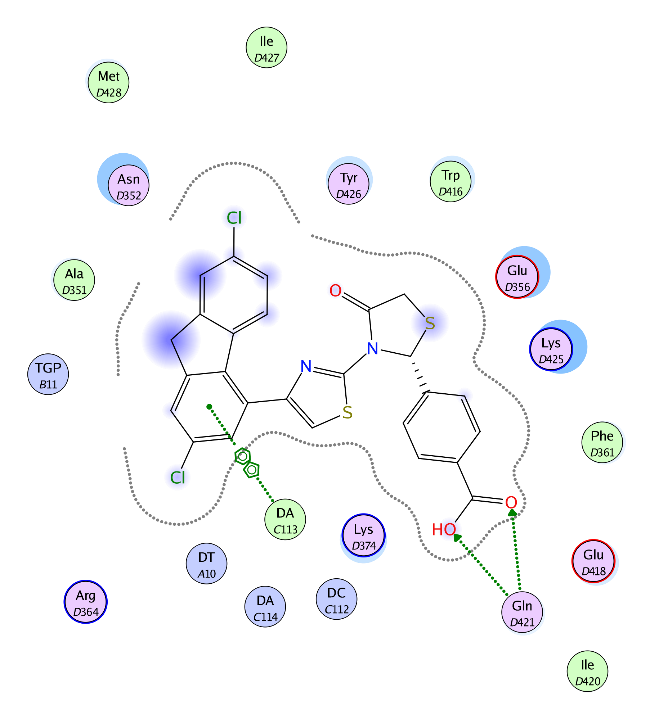 | **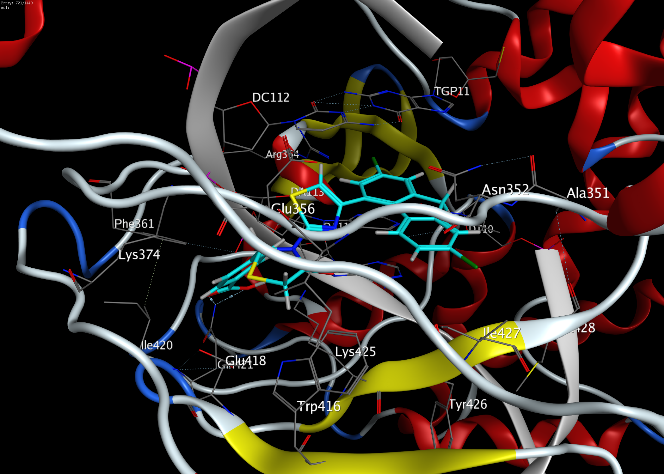** |
| 5i  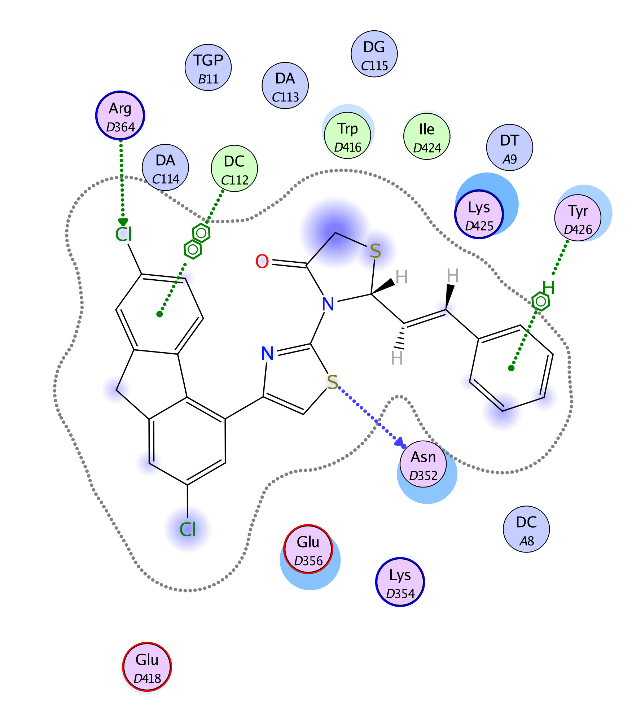 | **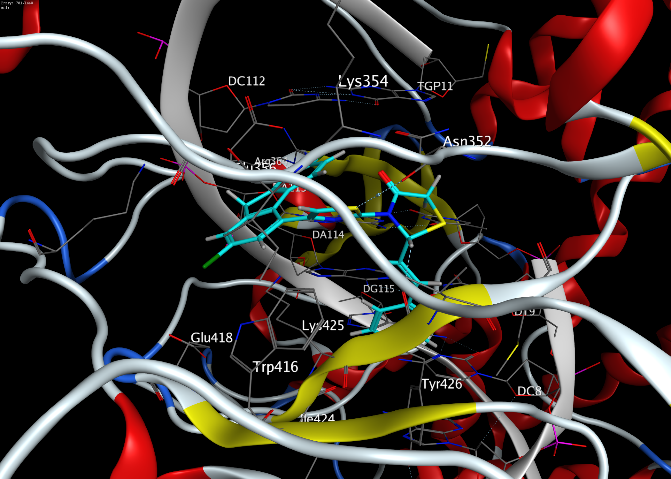** |
| 5j  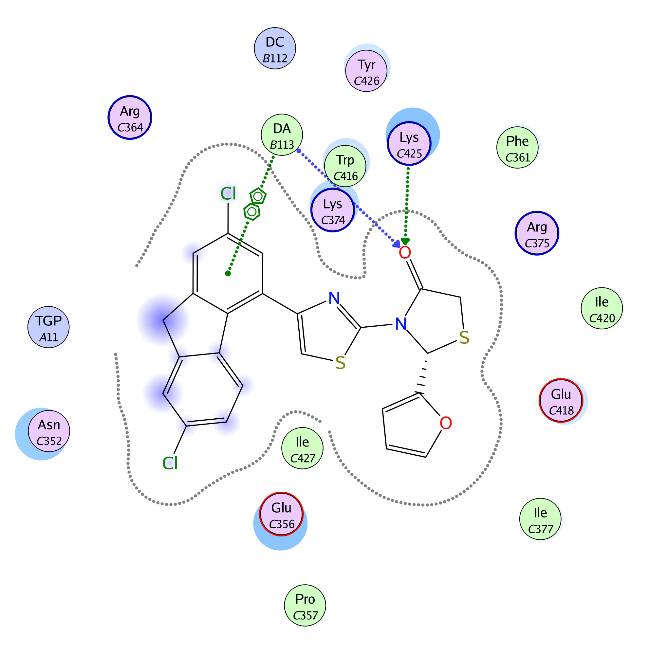 | **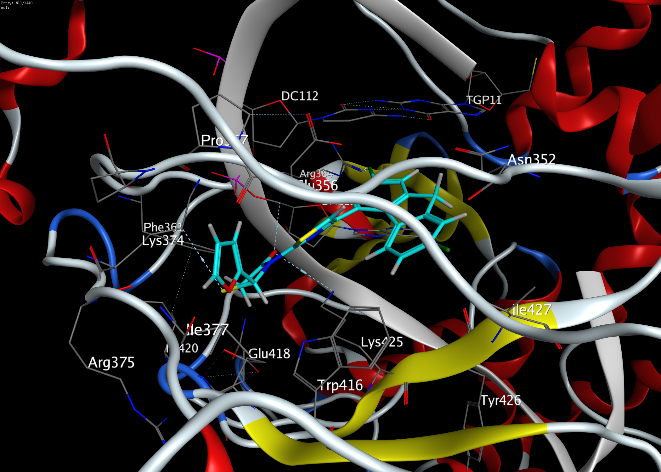** |
| 5k  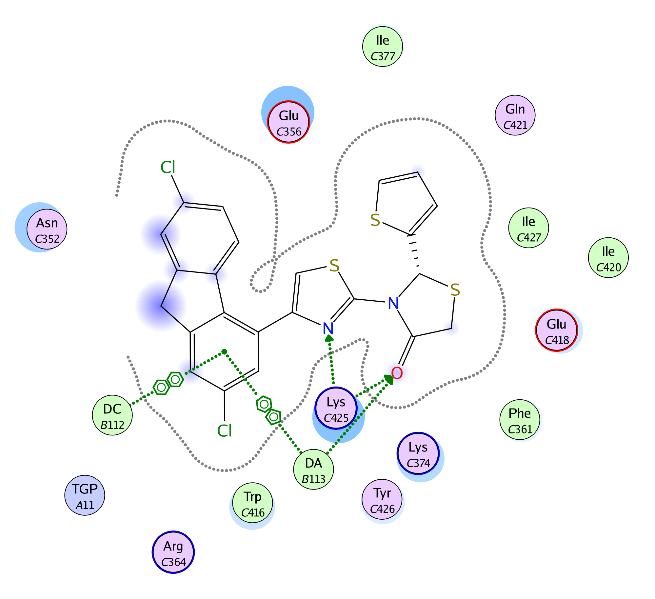 | **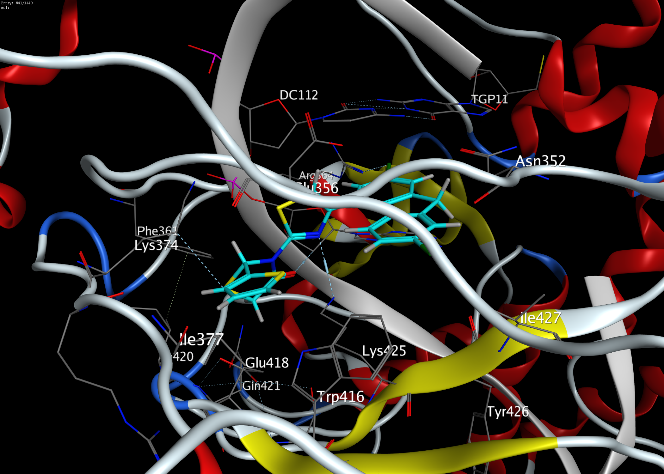** |
| 5m  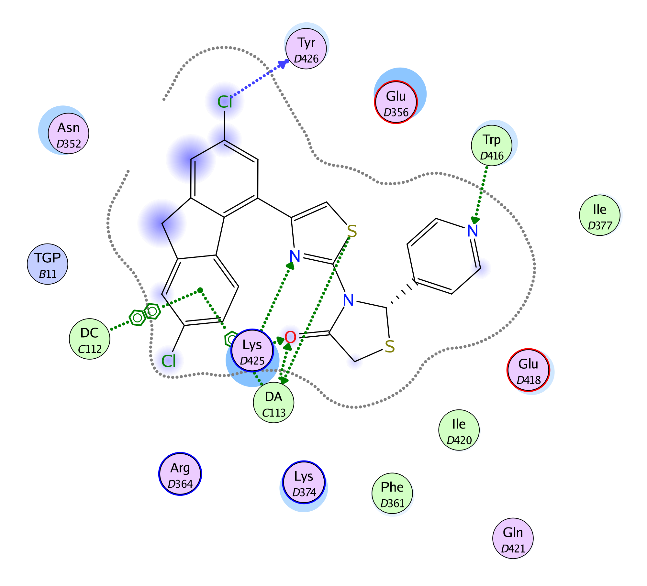 | **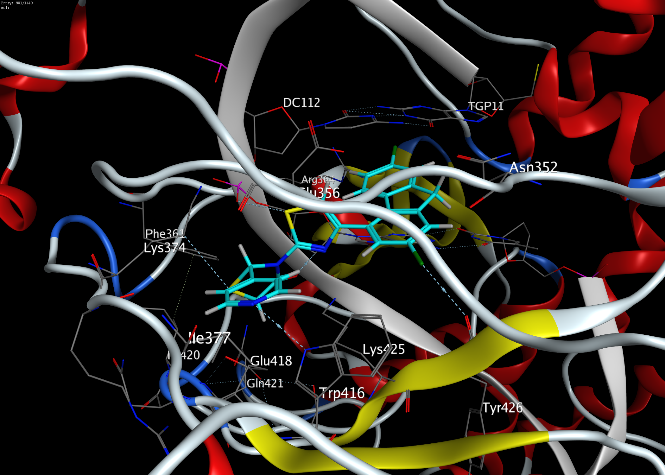** |
| 5n  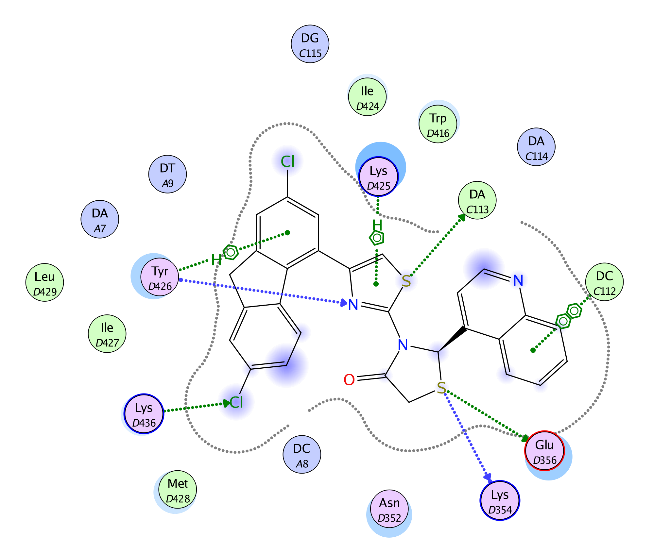 | **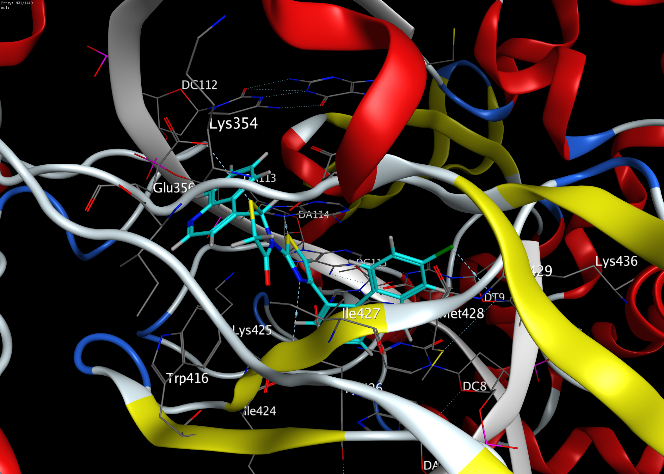** |
| 6a  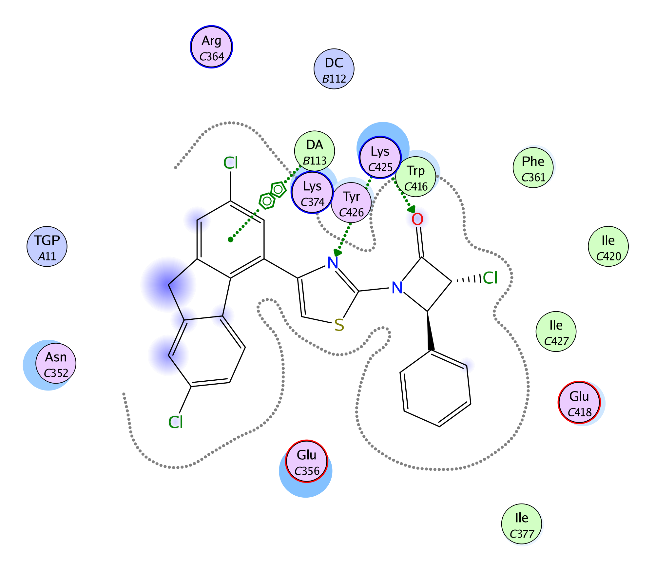 | **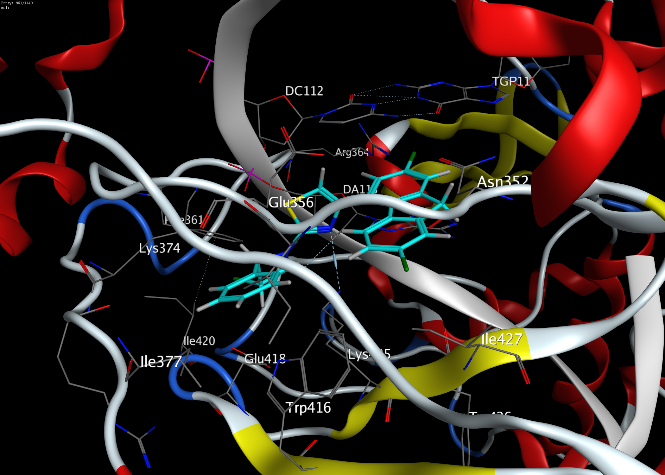** |
| 6b  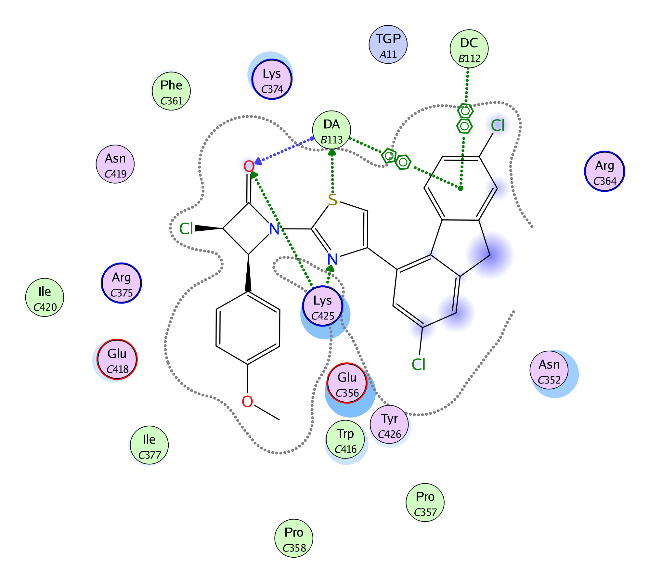 | **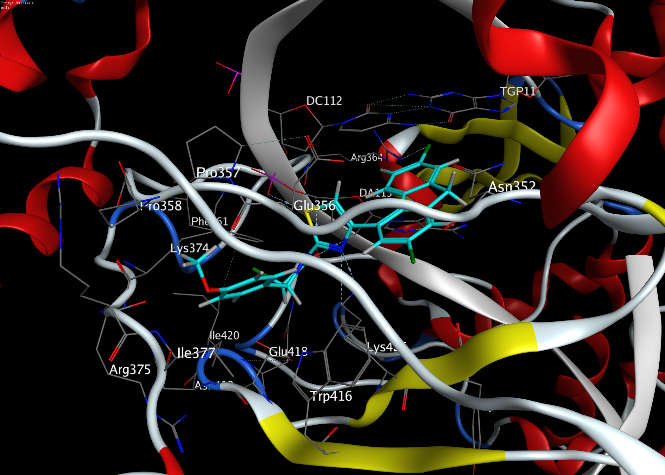** |
| 6c  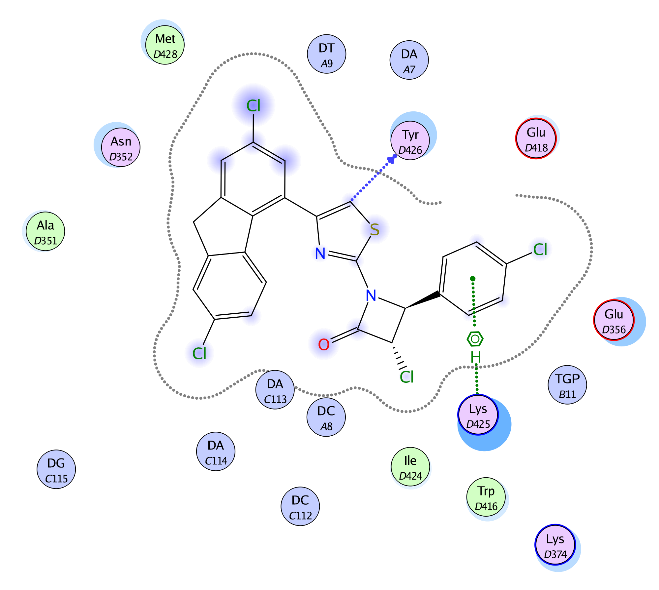 | **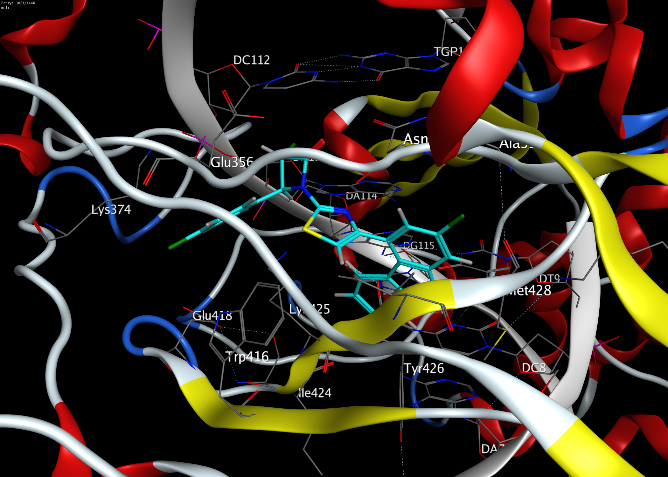** |
| 6d  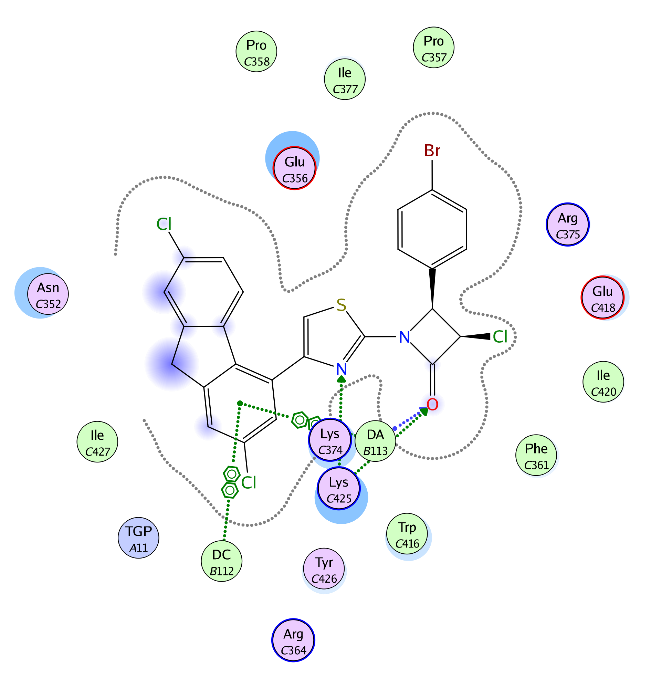 | **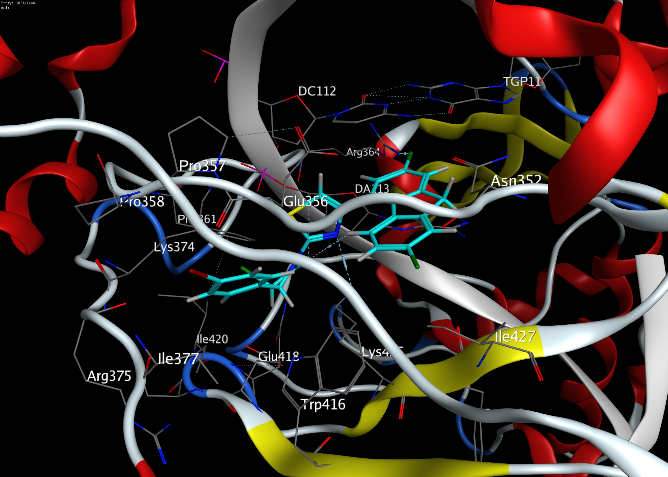** |
| 6f  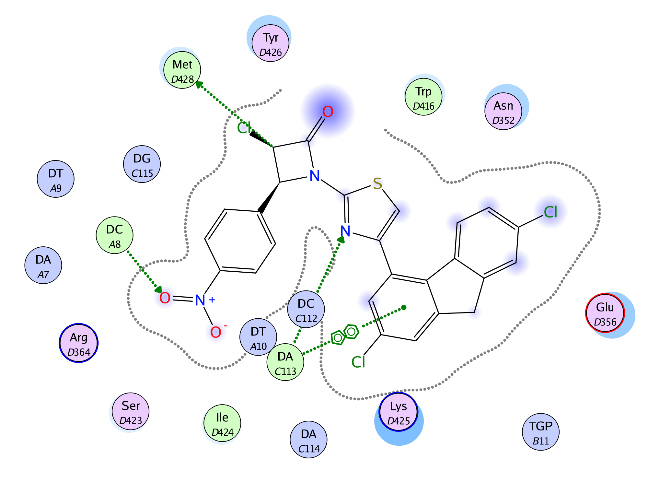 | **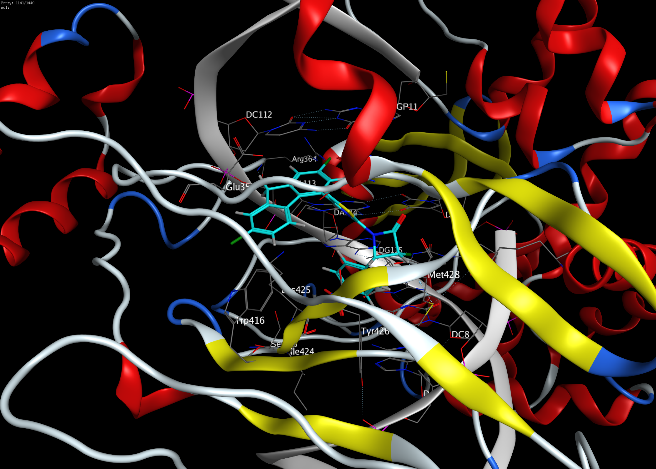** |
| 6g  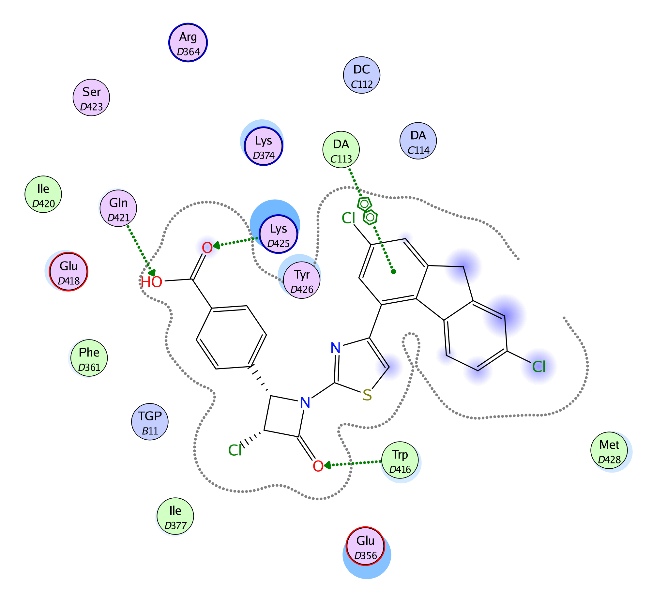 | **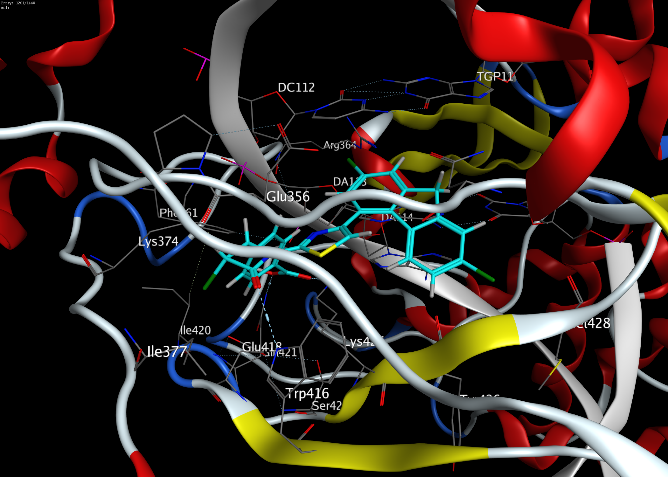** |
| 6i  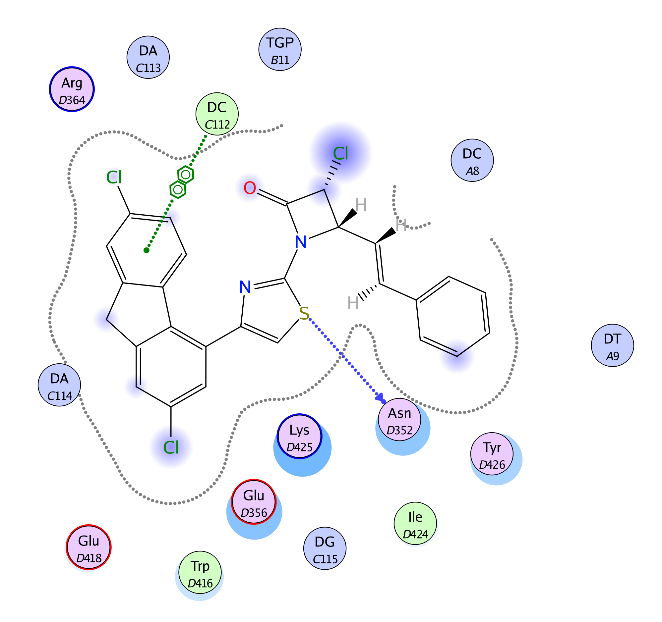 | **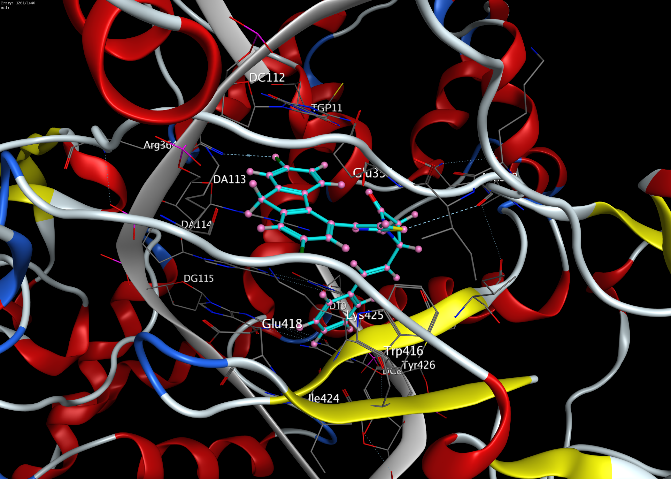** |
| 6j  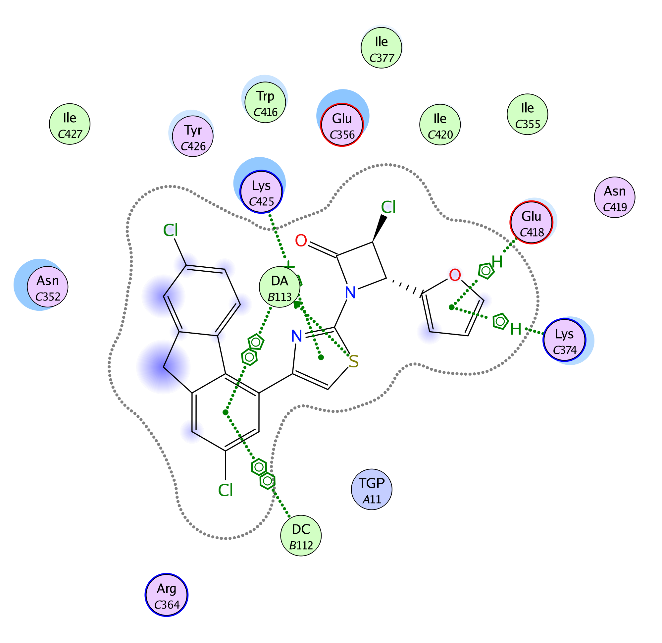 | **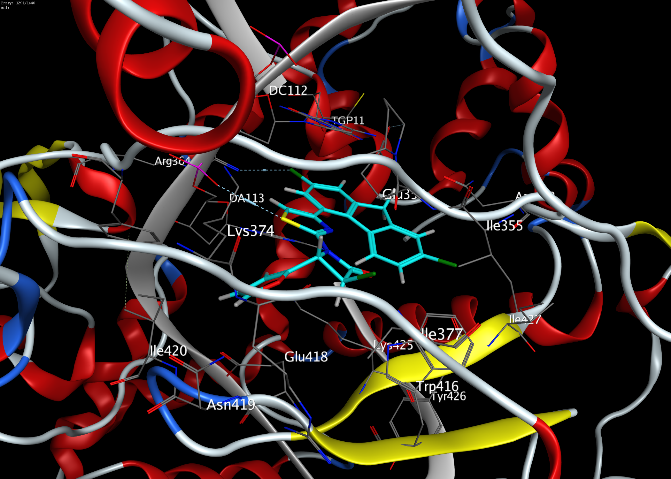** |
| 6l  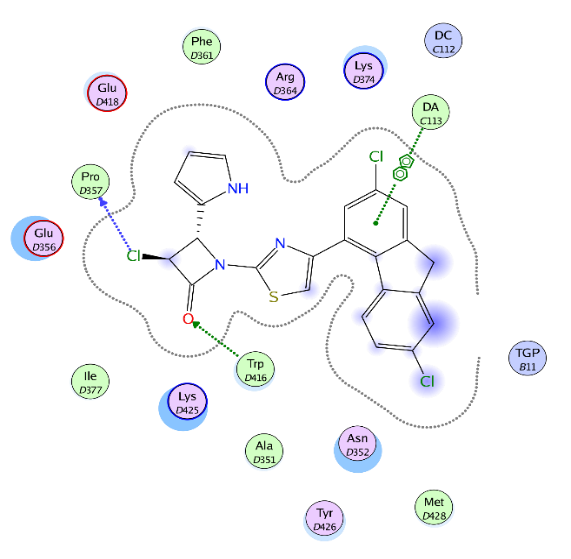 | **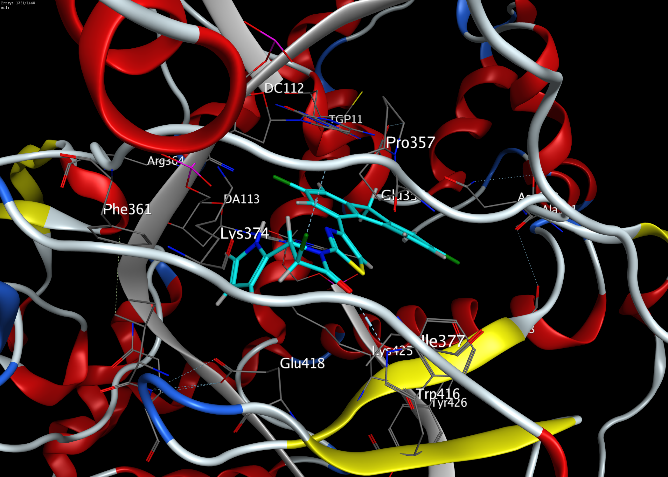** |
| 6m  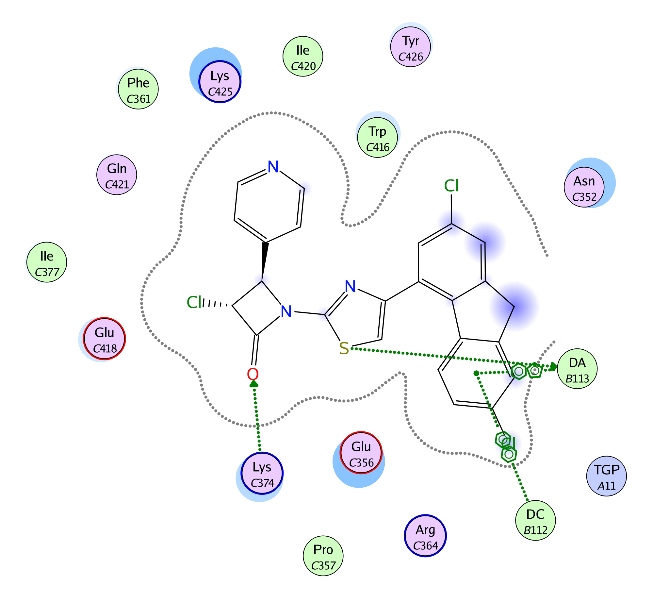 | **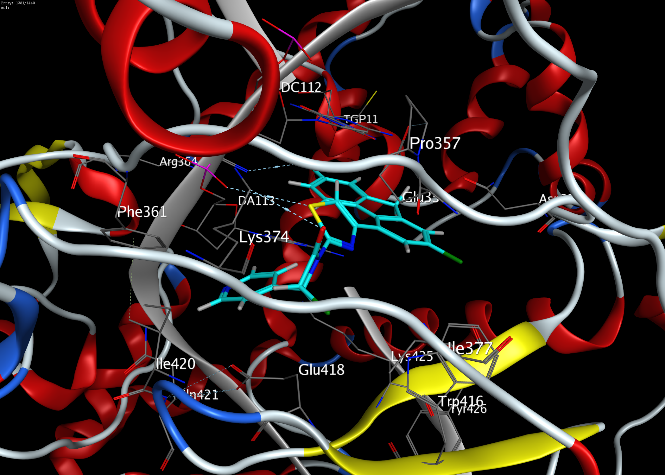** |
| 6n  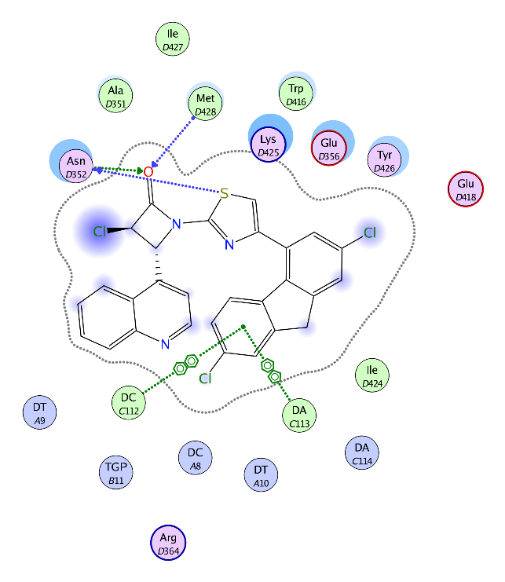 | **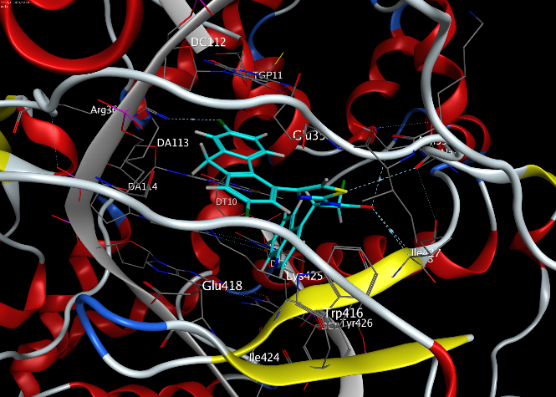** |
